# Supplementary material for: Elements of organisation of integrated maternity care and their associations with outcomes: a scoping review protocol
Source: BMJ Open. 2024 Jan 4;14(1):e075344. doi: 10.1136/bmjopen-2023-075344 (PMC10773375; doi:10.1136/bmjopen-2023-075344)
Supplement: Supplementary data [file bmjopen-2023-075344supp002.pdf]

VU VRIJE UNIVERSITEIT AMSTERDAM

Liebregts search strategies 4 August 2022

Search Strategy for PubMed (4 August 2022)

Search	Query	Results
**#6**	#4 NOT ("Developing Countries"[Mesh] OR "developing countr*"[tiab] OR "developing nation*"[tiab] OR "developing population*"[tiab] OR "developing econom*"[tiab] OR "undeveloped countr*"[tiab] OR "undeveloped nation*"[tiab] OR "undeveloped economy"[tiab] OR "undeveloped economies"[tiab] OR "least developed countr*"[tiab] OR "least developed nation*"[tiab] OR "least developed economy"[tiab] OR "least developed economies"[tiab] OR "less-developed countr*"[tiab] OR "less-developed nation*"[tiab] OR "less-developed population"[tiab] OR "less-developed populations"[tiab] OR "less-developed econom*"[tiab] OR "lesser developed countr*"[tiab] OR "lesser developed nation*"[tiab] OR "lesser developed population"[tiab] OR "lesser developed populations"[tiab] OR "lesser developed economy"[tiab] OR "lesser developed economies"[tiab] OR "under-developed countr*"[tiab] OR "under-developed nation*"[tiab] OR "underdeveloped countr*"[tiab] OR "underdeveloped nation*"[tiab] OR "underdeveloped population*"[tiab] OR "underdeveloped econom*"[tiab] OR "low income countr*"[tiab] OR "middle income countr*"[tiab] OR "low income nation*"[tiab] OR "middle income nation*"[tiab] OR "low income population*"[tiab] OR "middle income population*"[tiab] OR "low income econom*"[tiab] OR "middle income econom*"[tiab] OR "lower income countr*"[tiab] OR "lower income nation*"[tiab] OR "lower income population*"[tiab] OR "lower income economy"[tiab] OR "lower income economies"[tiab] OR "resource limited"[tiab] OR "low resource countr*"[tiab] OR "lower resource countr*"[tiab] OR "low resource nation*"[tiab] OR "low resource population*"[tiab] OR "low resource economy"[tiab] OR "low resource economies"[tiab] OR "underserved countr*"[tiab] OR "underserved nation*"[tiab] OR "underserved population*"[tiab] OR "underserved economy"[tiab] OR "underserved economies"[tiab] OR "under-served country"[tiab] OR "under-served countries"[tiab] OR "under-served nation"[tiab] OR "under-served nations"[tiab] OR "under-served population"[tiab] OR "under-served populations"[tiab] OR "underserved economy"[tiab] OR "underserved economies"[tiab] OR "deprived countr*"[tiab] OR "deprived nation"[tiab] OR "deprived nations"[tiab] OR "deprived population*"[tiab] OR "deprived economy"[tiab] OR "deprived economies"[tiab] OR "poor countr*"[tiab] OR "poor nation*"[tiab] OR "poor population*"[tiab] OR "poor econom*"[tiab] OR "poorer countr*"[tiab] OR "poorer nation*"[tiab] OR "poorer population*"[tiab] OR "poorer econom*"[tiab] OR "lmic"[tiab] OR "lmics"[tiab] OR "lami"[tiab] OR "transitional countr*"[tiab] OR "transitional nation"[tiab] OR "transitional nations"[tiab] OR "transitional econom*"[tiab] OR "transition countr*"[tiab] OR "transition nation*"[tiab] OR "transition econom*"[tiab] OR "low resource setting*"[tiab] OR "lower resource setting*"[tiab] OR "middle resource setting*"[tiab] OR "Third World*"[tiab]) Filters**:** from 2012 - 2022	**28,250**

VRIJE UNIVERSITEIT AMSTERDAM

**#5**	#4 NOT ("Developing Countries"[Mesh] OR "developing countr*"[tiab] OR "developing nation*"[tiab] OR "developing population*"[tiab] OR "developing econom*"[tiab] OR "undeveloped countr*"[tiab] OR "undeveloped nation*"[tiab] OR "undeveloped economy"[tiab] OR "undeveloped economies"[tiab] OR "least developed countr*"[tiab] OR "least developed nation*"[tiab] OR "least developed economy"[tiab] OR "least developed economies"[tiab] OR "less-developed countr*"[tiab] OR "less-developed nation*"[tiab] OR "less-developed population"[tiab] OR "less-developed populations"[tiab] OR "less-developed econom*"[tiab] OR "lesser developed countr*"[tiab] OR "lesser developed nation*"[tiab] OR "lesser developed population"[tiab] OR "lesser developed populations"[tiab] OR "lesser developed economy"[tiab] OR "lesser developed economies"[tiab] OR "under-developed countr*"[tiab] OR "under-developed nation*"[tiab] OR "underdeveloped countr*"[tiab] OR "underdeveloped nation*"[tiab] OR "underdeveloped population*"[tiab] OR "underdeveloped econom*"[tiab] OR "low income countr*"[tiab] OR "middle income countr*"[tiab] OR "low income nation*"[tiab] OR "middle income nation*"[tiab] OR "low income population*"[tiab] OR "middle income population*"[tiab] OR "low income econom*"[tiab] OR "middle income econom*"[tiab] OR "lower income countr*"[tiab] OR "lower income nation*"[tiab] OR "lower income population*"[tiab] OR "lower income economy"[tiab] OR "lower income economies"[tiab] OR "resource limited"[tiab] OR "low resource countr*"[tiab] OR "lower resource countr*"[tiab] OR "low resource nation*"[tiab] OR "low resource population*"[tiab] OR "low resource economy"[tiab] OR "low resource economies"[tiab] OR "underserved countr*"[tiab] OR "underserved nation*"[tiab] OR "underserved population*"[tiab] OR "underserved economy"[tiab] OR "underserved economies"[tiab] OR "under-served country"[tiab] OR "under-served countries"[tiab] OR "under-served nation"[tiab] OR "under-served nations"[tiab] OR "under-served population"[tiab] OR "under-served populations"[tiab] OR "underserved economy"[tiab] OR "underserved economies"[tiab] OR "deprived countr*"[tiab] OR "deprived nation"[tiab] OR "deprived nations"[tiab] OR "deprived population*"[tiab] OR "deprived economy"[tiab] OR "deprived economies"[tiab] OR "poor countr*"[tiab] OR "poor nation*"[tiab] OR "poor population*"[tiab] OR "poor econom*"[tiab] OR "poorer countr*"[tiab] OR "poorer nation*"[tiab] OR "poorer population*"[tiab] OR "poorer econom*"[tiab] OR "lmic"[tiab] OR "lmics"[tiab] OR "lami"[tiab] OR "transitional countr*"[tiab] OR "transitional nation"[tiab] OR "transitional nations"[tiab] OR "transitional econom*"[tiab] OR "transition countr*"[tiab] OR "transition nation*"[tiab] OR "transition econom*"[tiab] OR "low resource setting*"[tiab] OR "lower resource setting*"[tiab] OR "middle resource setting*"[tiab] OR "Third World*"[tiab])	**58,807**
**#4**	#1 AND #2 AND #3	**63,047**
**#3**	"preconception care"[MeSH Terms] OR "prenatal care"[MeSH Terms] OR "Pregnancy"[MeSH] OR "pregnant women"[MeSH Terms] OR "perinatal care"[MeSH Terms] OR "postnatal care"[MeSH Terms] OR "obstetrics"[MeSH Terms] OR "Midwifery"[Mesh] OR "prenatal care"[tiab] OR Antenatal[tiab] OR Preconception*[tiab] OR Maternal*[tiab] OR Maternity[tiab] OR postnat*[tiab] OR Postpartum[tiab] OR perinat*[tiab] OR Prenatal*[tiab] OR "birth care*"[tiab] OR Pregnan*[tiab] OR Midwive*[tiab] OR midwifery[tiab] OR obstetri*[tiab] OR Neonat*[tiab] OR Newborn*[tiab] OR "New born*"[tiab] OR "birth attendant*"[tiab] OR gynaecolog*[tiab] OR gynecolog*[tiab] OR "natal care*"[tiab]	**1,724,086**

**#2**	"maternal health"[MeSH Terms] OR "Pregnancy Outcome"[MeSH] OR "Pregnancy Complications"[Mesh] OR "infant health"[MeSH Terms] OR "Infant Mortality"[Mesh] OR "Morbidity"[Mesh] OR "Mortality"[Mesh] OR "Obstetric Labor Complications"[Mesh] OR "maternal health"[tiab] OR "infant health"[tiab] OR "neonatal health"[tiab] OR "neonate health"[tiab] OR "complications"[sb] OR "complicat*"[tiab] OR "adverse effects"[sb] OR "adverse effect*"[tiab] OR "adverse affect*"[tiab] OR "adversely affect*"[tiab] OR "side effect*"[tiab] OR "side affect*"[tiab] OR "adverse event*"[tiab] OR morbid*[tiab] OR death*[tiab] OR mortalit*[tiab] OR injur*[tiab] OR "adverse outcome*"[tiab] OR "Patient Satisfaction"[MeSH] OR ((Patient*[tiab] OR client*[tiab] OR "Women"[Mesh] OR Woman*[tiab] OR Women*[tiab]) AND (experience*[tiab] OR satisfaction*[tiab])) OR (("health personnel"[MeSH Terms] OR "Nurse Midwives"[Mesh] OR "Obstetrics"[Mesh] OR midwife*[tiab] OR midwiv*[tiab] OR Nurse*[tiab] OR "birth attendant*"[tiab] OR Obstetrician*[tiab] OR gynaecolog*[tiab] OR gynecolog*[tiab] Caregiver*[tiab] OR "care giver*"[tiab] OR Provider*[tiab] OR "health personnel"[tiab] OR "personnel health"[tiab]) AND (experience*[tiab] OR satisfaction*)) OR spending*[tiab] OR expenditure*[tiab] OR cost*[tiab] OR "Referral and Consultation"[Mesh] OR "patient transfer"[MeSH Terms] OR "Medicalization"[Mesh] OR Referral*[tiab] OR Intervention*[tiab] OR Transfer*[tiab] OR medicalization*[tiab] OR medicalisation*[tiab]	**7,660,615**
**#1**	"Intersectoral Collaboration"[Mesh] OR "Models, Organizational"[Mesh] OR "Delivery of Health Care, Integrated"[Mesh] OR "Organization and Administration"[Mesh] OR "integrated care*"[tiab] OR "integrated maternity care*"[tiab] OR "integration of care*"[tiab] OR "Integration of maternity care"[tiab] OR "transmural care"[tiab] OR "continuity of care"[tiab] OR "continuum of care"[tiab] OR "model of care*"[tiab] OR "models of care*"[tiab] OR "care model*"[tiab] OR "care system*"[tiab] OR "risk selection*"[tiab] OR "Shared care"[tiab] OR "Joint care"[tiab] OR "Collaborative care"[tiab] OR Interprofessional*[tiab] OR "Inter professional*"[tiab] OR Collaboration*[tiab] OR Interdisciplinair*[tiab] OR Multidisciplinair[tiab] OR Cooperat*[tiab] OR "co oporat*"[tiab]	**1,952,242**

Search Strategy for Elsevier/Scopus (4 August 2022)

Search	Query	Results
**#7**	#6 AND 2012-2022	**13,145**
**#6**	#5 NOT (conference paper OR conference review)	**22,153**
**#5**	#4 NOT TITLE-ABS-KEY ("developing countr*" OR "developing nation*" OR "developing population*" OR "developing econom*" OR "undeveloped countr*" OR "undeveloped nation*" OR "undeveloped economy" OR "undeveloped economies" OR "least developed countr*" OR "least developed nation*" OR "least developed economy" OR "least developed economies" OR "less-developed countr*" OR "less-developed nation*" OR "less-developed population" OR "less-developed populations" OR "less-developed econom*" OR "lesser developed countr*" OR "lesser developed nation*" OR "lesser developed population" OR "lesser developed populations" OR "lesser developed economy" OR "lesser developed economies" OR "under-developed countr*" OR "under-developed nation*" OR "underdeveloped countr*" OR "underdeveloped nation*" OR "underdeveloped population*" OR "underdeveloped econom*" OR "low income countr*" OR "middle income countr*" OR "low income nation*" OR "middle income nation*" OR "low income population*" OR "middle income population*" OR "low income econom*" OR "middle income econom*" OR "lower income countr*" OR "lower income	**22,727**

Liebregts J, *et al. BMJ Open* 2024; 14:e075344. doi: 10.1136/bmjopen-2023-075344

	nation*" OR "lower income population*" OR "lower income economy" OR "lower income economies" OR "resource limited" OR "low resource countr*" OR "lower resource countr*" OR "low resource nation*" OR "low resource population*" OR "low resource economy" OR "low resource economies" OR "underserved countr*" OR "underserved nation*" OR "underserved population*" OR "underserved economy" OR "underserved economies" OR "under-served country" OR "under-served countries" OR "under-served nation" OR "under-served nations" OR "under-served population" OR "under-served populations" OR "underserved economy" OR "underserved economies" OR "deprived countr*" OR "deprived nation" OR "deprived nations" OR "deprived population*" OR "deprived economy" OR "deprived economies" OR "poor countr*" OR "poor nation*" OR "poor population*" OR "poor econom*" OR "poorer countr*" OR "poorer nation*" OR "poorer population*" OR "poorer econom*" OR "lmic" OR "lmics" OR "lami" OR "transitional countr*" OR "transitional nation" OR "transitional nations" OR "transitional econom*" OR "transition countr*" OR "transition nation*" OR "transition econom*" OR "low resource setting*" OR "lower resource setting*" OR "middle resource setting*" OR "Third World*")	
**#4**	#1 AND #2 AND #3	**427,512**
**#3**	TITLE-ABS-KEY ("prenatal care" OR Antenatal OR Preconception* OR Maternal* OR Maternity OR postnat* OR Postpartum OR perinat* OR Prenatal* OR "birth care*" OR Pregnan* OR Midwive* OR midwifery OR obstetri* OR Neonat* OR Newborn* OR "New born*" OR "birth attendant*" OR gynaecolog* OR gynecolog* OR "natal care*")	**25,753**
**#2**	TITLE-ABS-KEY ("maternal health" OR "infant health" OR "neonatal health" OR "neonate health" OR "complicat*" OR "adverse effect*" OR "adverse affect*" OR "adversely affect*" OR "side effect*" OR "side affect*" OR "adverse event*" OR morbid* OR death* OR mortalit* OR injur* OR "adverse outcome*" OR ((Patient* OR client* OR Woman* OR Women*) AND (experience* OR satisfaction*)) OR ((midwife* OR midwiv* OR Nurse* OR "birth attendant*" OR Obstetrician* OR gynaecolog* OR gynecolog* Caregiver* OR "care giver*" OR Provider* OR "health personnel" OR "personnel health") AND (experience* OR satisfaction*)) OR spending* OR expenditure* OR cost* OR Referral* OR Intervention* OR Transfer* OR medicalization* OR medicalisation*)	**2,516,011**
**#1**	TITLE-ABS-KEY ("integrated care*" OR "integrated maternity care*" OR "integration of care*" OR "Integration of maternity care" OR "transmural care" OR "continuity of care" OR "continuum of care" OR "model of care*" OR "models of care*" OR "care model*" OR "care system*" OR "risk selection*" OR "Shared care" OR "Joint care" OR "Collaborative care" OR Interprofessional* OR "Inter professional*" OR Collaboration* OR Interdisciplinair* OR Multidisciplinair OR Cooperat* OR "co oporat*")	**15,178,910**

Search Strategy for Ebsco/APA PsycINFO (4 August 2022)

Search	Query	Results
**#5**	S4 NOT (DE "Developing Countries" OR TI("developing countr*" OR "developing nation*" OR "developing population*" OR "developing econom*" OR "undeveloped countr*" OR "undeveloped nation*" OR "undeveloped economy" OR "undeveloped economies" OR "least developed countr*" OR "least developed nation*" OR "least developed economy" OR "least developed economies" OR "less-developed countr*" OR "less-developed nation*" OR "less-developed population" OR "less-developed populations" OR "less-developed econom*" OR "lesser developed countr*" OR "lesser developed nation*" OR "lesser developed population" OR "lesser developed populations" OR "lesser developed economy" OR "lesser developed economies" OR	**1,019**

	"under-developed countr*" OR "under-developed nation*" OR "underdeveloped countr*" OR "underdeveloped nation*" OR "underdeveloped population*" OR "underdeveloped econom*" OR "low income countr*" OR "middle income countr*" OR "low income nation*" OR "middle income nation*" OR "low income population*" OR "middle income population*" OR "low income econom*" OR "middle income econom*" OR "lower income countr*" OR "lower income nation*" OR "lower income population*" OR "lower income economy" OR "lower income economies" OR "resource limited" OR "low resource countr*" OR "lower resource countr*" OR "low resource nation*" OR "low resource population*" OR "low resource economy" OR "low resource economies" OR "underserved countr*" OR "underserved nation*" OR "underserved population*" OR "underserved economy" OR "underserved economies" OR "under-served country" OR "under-served countries" OR "under-served nation" OR "under-served nations" OR "under-served population" OR "under-served populations" OR "underserved economy" OR "underserved economies" OR "deprived countr*" OR "deprived nation" OR "deprived nations" OR "deprived population*" OR "deprived economy" OR "deprived economies" OR "poor countr*" OR "poor nation*" OR "poor population*" OR "poor econom*" OR "poorer countr*" OR "poorer nation*" OR "poorer population*" OR "poorer econom*" OR "lmic" OR "lmics" OR "lami" OR "transitional countr*" OR "transitional nation" OR "transitional nations" OR "transitional econom*" OR "transition countr*" OR "transition nation*" OR "transition econom*" OR "low resource setting*" OR "lower resource setting*" OR "middle resource setting*" OR "Third World*") OR AB("developing countr*" OR "developing nation*" OR "developing population*" OR "developing econom*" OR "undeveloped countr*" OR "undeveloped nation*" OR "undeveloped economy" OR "undeveloped economies" OR "least developed countr*" OR "least developed nation*" OR "least developed economy" OR "least developed economies" OR "less-developed countr*" OR "less-developed nation*" OR "less-developed population" OR "less-developed populations" OR "less-developed econom*" OR "lesser developed countr*" OR "lesser developed nation*" OR "lesser developed population" OR "lesser developed populations" OR "lesser developed economy" OR "lesser developed economies" OR "under-developed countr*" OR "under-developed nation*" OR "underdeveloped countr*" OR "underdeveloped nation*" OR "underdeveloped population*" OR "underdeveloped econom*" OR "low income countr*" OR "middle income countr*" OR "low income nation*" OR "middle income nation*" OR "low income population*" OR "middle income population*" OR "low income econom*" OR "middle income econom*" OR "lower income countr*" OR "lower income nation*" OR "lower income population*" OR "lower income economy" OR "lower income economies" OR "resource limited" OR "low resource countr*" OR "lower resource countr*" OR "low resource nation*" OR "low resource population*" OR "low resource economy" OR "low resource economies" OR "underserved countr*" OR "underserved nation*" OR "underserved population*" OR "underserved economy" OR "underserved economies" OR "under-served country" OR "under-served countries" OR "under-served nation" OR "under-served nations" OR "under-served population" OR "under-served populations" OR "underserved economy" OR "underserved economies" OR "deprived countr*" OR "deprived nation" OR "deprived nations" OR "deprived population*" OR "deprived economy" OR "deprived economies" OR "poor countr*" OR "poor nation*" OR "poor population*" OR "poor econom*" OR "poorer countr*" OR "poorer nation*" OR "poorer population*" OR "poorer econom*" OR "lmic" OR "lmics" OR "lami" OR "transitional countr*" OR "transitional nation" OR "transitional nations" OR "transitional econom*" OR "transition countr*" OR "transition nation*" OR	

	"transition econom*" OR "low resource setting*" OR "lower resource setting*" OR "middle resource setting*" OR "Third World*") OR KW("developing countr*" OR "developing nation*" OR "developing population*" OR "developing econom*" OR "undeveloped countr*" OR "undeveloped nation*" OR "undeveloped economy" OR "undeveloped economies" OR "least developed countr*" OR "least developed nation*" OR "least developed economy" OR "least developed economies" OR "less-developed countr*" OR "less-developed nation*" OR "less-developed population" OR "less-developed populations" OR "less-developed econom*" OR "lesser developed countr*" OR "lesser developed nation*" OR "lesser developed population" OR "lesser developed populations" OR "lesser developed economy" OR "lesser developed economies" OR "under-developed countr*" OR "under-developed nation*" OR "underdeveloped countr*" OR "underdeveloped nation*" OR "underdeveloped population*" OR "underdeveloped econom*" OR "low income countr*" OR "middle income countr*" OR "low income nation*" OR "middle income nation*" OR "low income population*" OR "middle income population*" OR "low income econom*" OR "middle income econom*" OR "lower income countr*" OR "lower income nation*" OR "lower income population*" OR "lower income economy" OR "lower income economies" OR "resource limited" OR "low resource countr*" OR "lower resource countr*" OR "low resource nation*" OR "low resource population*" OR "low resource economy" OR "low resource economies" OR "underserved countr*" OR "underserved nation*" OR "underserved population*" OR "underserved economy" OR "underserved economies" OR "under-served country" OR "under-served countries" OR "under-served nation" OR "under-served nations" OR "under-served population" OR "under-served populations" OR "underserved economy" OR "underserved economies" OR "deprived countr*" OR "deprived nation" OR "deprived nations" OR "deprived population*" OR "deprived economy" OR "deprived economies" OR "poor countr*" OR "poor nation*" OR "poor population*" OR "poor econom*" OR "poorer countr*" OR "poorer nation*" OR "poorer population*" OR "poorer econom*" OR "lmic" OR "lmics" OR "lami" OR "transitional countr*" OR "transitional nation" OR "transitional nations" OR "transitional econom*" OR "transition countr*" OR "transition nation*" OR "transition econom*" OR "low resource setting*" OR "lower resource setting*" OR "middle resource setting*" OR "Third World*"))<br>2012 - 2022	
**#4**	#1 AND #2 AND #3	**1,695**
**#3**	DE "Prenatal Care" OR DE "Childbirth Training" OR DE "Pregnancy" OR DE "Adolescent Pregnancy" OR OR DE "Primipara" OR DE "Perinatal Period" OR DE "Postnatal Period" OR DE "Obstetrics" OR DE "Caesarean Birth" OR DE "Midwifery" OR TI("prenatal care" OR Antenatal OR Preconception* OR Maternal* OR Maternity OR postnat* OR Postpartum OR perinat* OR Prenatal* OR "birth care*" OR Pregnan* OR Midwive* OR midwifery OR obstetri* OR Neonat* OR Newborn* OR "New born*" OR "birth attendant*" OR gynaecolog* OR gynecolog* OR "natal care*") OR AB("prenatal care" OR Antenatal OR Preconception* OR Maternal* OR Maternity OR postnat* OR Postpartum OR perinat* OR Prenatal* OR "birth care*" OR Pregnan* OR Midwive* OR midwifery OR obstetri* OR Neonat* OR Newborn* OR "New born*" OR "birth attendant*" OR gynaecolog* OR gynecolog* OR "natal care*") OR KW("prenatal care" OR Antenatal OR Preconception* OR Maternal* OR Maternity OR postnat* OR Postpartum OR perinat* OR Prenatal* OR "birth care*" OR Pregnan* OR Midwive* OR midwifery OR obstetri* OR Neonat* OR Newborn* OR "New born*" OR "birth attendant*" OR gynaecolog* OR gynecolog* OR "natal care*")	**169,395**

VRIJE UNIVERSITEIT AMSTERDAM

**#2**	DE "Pregnancy Outcomes" OR DE "Birth" OR DE "Induced Abortion" OR DE "Obstetrical Complications" OR DE "Spontaneous Abortion" OR DE "Morbidity" OR DE "Premorbidity" OR DE "Mortality Rate" OR DE "Mortality Risk" OR DE "Client Satisfaction" OR DE "Client Transfer" OR (("Women"[Mesh] OR "health personnel"[MeSH Terms] OR "Nurse Midwives"[Mesh] OR "Obstetrics"[Mesh]) AND TI(experience* OR satisfaction*) OR AB(experience* OR satisfaction*) OR KW(experience* OR satisfaction*)) OR TI("maternal health" OR "infant health" OR "neonatal health" OR "neonate health" OR "complicat*" OR OR "adverse effect*" OR "adverse affect*" OR "adversely affect*" OR "side effect*" OR "side affect*" OR "adverse event*" OR morbid* OR death* OR mortalit* OR injur* OR "adverse outcome*" OR spending* OR expenditure* OR cost* OR Referral* OR Intervention* OR Transfer* OR medicalization* OR medicalisation*) OR AB("maternal health" OR "infant health" OR "neonatal health" OR "neonate health" OR "complicat*" OR OR "adverse effect*" OR "adverse affect*" OR "adversely affect*" OR "side effect*" OR "side affect*" OR "adverse event*" OR morbid* OR death* OR mortalit* OR injur* OR "adverse outcome*" OR spending* OR expenditure* OR cost* OR Referral* OR Intervention* OR Transfer* OR medicalization* OR medicalisation*) OR KW("maternal health" OR "infant health" OR "neonatal health" OR "neonate health" OR "complicat*" OR OR "adverse effect*" OR "adverse affect*" OR "adversely affect*" OR "side effect*" OR "side affect*" OR "adverse event*" OR morbid* OR death* OR mortalit* OR injur* OR "adverse outcome*" OR spending* OR expenditure* OR cost* OR Referral* OR Intervention* OR Transfer* OR medicalization* OR medicalisation*) OR TI((Patient* OR client* OR OR Woman* OR Women* OR midwife* OR midwiv* OR Nurse* OR "birth attendant*" OR Obstetrician* OR gynaecolog* OR gynecolog* Caregiver* OR "care giver*" OR Provider* OR "health personnel" OR "personnel health") N3 (experience* OR satisfaction*)) OR AB((Patient* OR client* OR OR Woman* OR Women* OR midwife* OR midwiv* OR Nurse* OR "birth attendant*" OR Obstetrician* OR gynaecolog* OR gynecolog* Caregiver* OR "care giver*" OR Provider* OR "health personnel" OR "personnel health") N3 (experience* OR satisfaction*)) OR KW((Patient* OR client* OR OR Woman* OR Women* OR midwife* OR midwiv* OR Nurse* OR "birth attendant*" OR Obstetrician* OR gynaecolog* OR gynecolog* Caregiver* OR "care giver*" OR Provider* OR "health personnel" OR "personnel health") N3 (experience* OR satisfaction*))	**965,418**
**#1**	DE "Integrated Services" OR TI("integrated care*" OR "integrated maternity care*" OR "integration of care*" OR "Integration of maternity care" OR "transmural care" OR "continuity of care" OR "continuum of care" OR "model of care*" OR "models of care*" OR "care model*" OR "care system*" OR "risk selection*" OR "Shared care" OR "Joint care" OR "Collaborative care" OR Interprofessional* OR "Inter professional*" OR Collaboration* OR Interdisciplinair* OR Multidisciplinair OR Cooperat* OR "co oporat*") OR AB("integrated care*" OR "integrated maternity care*" OR "integration of care*" OR "Integration of maternity care" OR "transmural care" OR "continuity of care" OR "continuum of care" OR "model of care*" OR "models of care*" OR "care model*" OR "care system*" OR "risk selection*" OR "Shared care" OR "Joint care" OR "Collaborative care" OR Interprofessional* OR "Inter professional*" OR Collaboration* OR Interdisciplinair* OR Multidisciplinair OR Cooperat* OR "co oporat*") OR KW("integrated care*" OR "integrated maternity care*" OR "integration of care*" OR "Integration of maternity care" OR "transmural care" OR "continuity of care" OR "continuum of care" OR "model of care*" OR "models of care*" OR "care model*" OR "care system*" OR "risk selection*" OR "Shared care" OR "Joint care" OR "Collaborative care"	**129,901**

Versie: 1

	OR Interprofessional* OR "Inter professional*" OR Collaboration* OR Interdisciplinair* OR Multidisciplinair OR Cooperat* OR "co oporat*")	

Search Strategy for Wiley/Cochrane Library (4 August 2022)

Search	Query	Results
**#6**	#5 AND 2012-2022	**2,112**
**#5**	#4 NOT ("developing countr*" OR "developing nation*" OR "developing population*" OR "developing econom*" OR "undeveloped countr*" OR "undeveloped nation*" OR "undeveloped economy" OR "undeveloped economies" OR "least developed countr*" OR "least developed nation*" OR "least developed economy" OR "least developed economies" OR "less-developed countr*" OR "less-developed nation*" OR "less-developed population" OR "less-developed populations" OR "less-developed econom*" OR "lesser developed countr*" OR "lesser developed nation*" OR "lesser developed population" OR "lesser developed populations" OR "lesser developed economy" OR "lesser developed economies" OR "under-developed countr*" OR "under-developed nation*" OR "underdeveloped countr*" OR "underdeveloped nation*" OR "underdeveloped population*" OR "underdeveloped econom*" OR "low income countr*" OR "middle income countr*" OR "low income nation*" OR "middle income nation*" OR "low income population*" OR "middle income population*" OR "low income econom*" OR "middle income econom*" OR "lower income countr*" OR "lower income nation*" OR "lower income population*" OR "lower income economy" OR "lower income economies" OR "resource limited" OR "low resource countr*" OR "lower resource countr*" OR "low resource nation*" OR "low resource population*" OR "low resource economy" OR "low resource economies" OR "underserved countr*" OR "underserved nation*" OR "underserved population*" OR "underserved economy" OR "underserved economies" OR "under-served country" OR "under-served countries" OR "under-served nation" OR "under-served nations" OR "under-served population" OR "under-served populations" OR "underserved economy" OR "underserved economies" OR "deprived countr*" OR "deprived nation" OR "deprived nations" OR "deprived population*" OR "deprived economy" OR "deprived economies" OR "poor countr*" OR "poor nation*" OR "poor population*" OR "poor econom*" OR "poorer countr*" OR "poorer nation*" OR "poorer population*" OR "poorer econom*" OR "lmic" OR "lmics" OR "lami" OR "transitional countr*" OR "transitional nation" OR "transitional nations" OR "transitional econom*" OR "transition countr*" OR "transition nation*" OR "transition econom*" OR "low resource setting*" OR "lower resource setting*" OR "middle resource setting*" OR "Third World*"):ti,ab,kw	**2,121**
**#4**	#1 AND #2 AND #3	**2,156**
**#3**	("prenatal care" OR Antenatal OR Preconception* OR Maternal* OR Maternity OR postnat* OR Postpartum OR perinat* OR Prenatal* OR "birth care*" OR Pregnan* OR Midwive* OR midwifery OR obstetri* OR Neonat* OR Newborn* OR "New born*" OR "birth attendant*" OR gynaecolog* OR gynecolog* OR "natal care*"):ti,ab,kw	**126,263**
**#2**	("maternal health" OR "infant health" OR "neonatal health" OR "neonate health" OR "complicat*" OR "adverse effect*" OR "adverse affect*" OR "adversely affect*" OR "side effect*" OR "side affect*" OR "adverse event*" OR morbid* OR death* OR mortalit* OR injur* OR "adverse outcome*" OR ((Patient* OR client* OR Woman* OR Women*) AND (experience* OR satisfaction*)) OR ((midwife* OR midwiv* OR Nurse* OR "birth attendant*" OR Obstetrician* OR gynaecolog* OR gynecolog* Caregiver* OR "care giver*" OR Provider* OR "health personnel" OR "personnel health") AND (experience* OR satisfaction*)) OR	**837,605**

VRIJE UNIVERSITEIT AMSTERDAM

	spending* OR expenditure* OR cost* OR Referral* OR Intervention* OR Transfer* OR medicalization* OR medicalisation*):ti,ab,kw	
**#1**	**(**"integrated care*" OR "integrated maternity care*" OR "integration of care*" OR "Integration of maternity care" OR "transmural care" OR "continuity of care" OR "continuum of care" OR "model of care*" OR "models of care*" OR "care model*" OR "care system*" OR "risk selection*" OR "Shared care" OR "Joint care" OR "Collaborative care" OR Interprofessional* OR "Inter professional*" OR Collaboration* OR Interdisciplinair* OR Multidisciplinair OR Cooperat* OR "co oporat*"):ti,ab,kw	**31,496**
