# Supplementary material for: Elements of organisation of integrated maternity care and their associations with outcomes: a scoping review protocol
Source: BMJ Open. 2024 Jan 4;14(1):e075344. doi: 10.1136/bmjopen-2023-075344 (PMC10773375; doi:10.1136/bmjopen-2023-075344)
Supplement: Supplementary data [file bmjopen-2023-075344supp003.pdf]

Supplementary Table 1. Draft Data Extraction Table.

Element of integrated maternity care	Outcomes					Levels RMIC								Level of evidence
Element of integrated maternity care	Outcome 1. Maternal and neonatal health	Outcome 2. Experiences of professionals	Outcome 3. Experiences of mothers	Outcome 4. Healthcare processes	Healthcare spending	Vertical – Horizontal organization:	Degree of integration	Clinical integration	Professional integration	Organizational integration:	System Integration	Functional enablers:	Normative enablers:	Level of evidence
e.g. mothercouncil														
e.g. multidisciplinary consultation														
