# Supplementary material for: Elements of organisation of integrated maternity care and their associations with outcomes: a scoping review protocol
Source: BMJ Open. 2024 Jan 4;14(1):e075344. doi: 10.1136/bmjopen-2023-075344 (PMC10773375; doi:10.1136/bmjopen-2023-075344)
Supplement: Author's manuscript [file bmjopen-2023-075344.draft_revisions.pdf]

BMJ Open is committed to open peer review. As part of this commitment we make the peer review history of every article we publish publicly available.

When an article is published we post the peer reviewers' comments and the authors' responses online. We also post the versions of the paper that were used during peer review. These are the versions that the peer review comments apply to.

The versions of the paper that follow are the versions that were submitted during the peer review process. They are not the versions of record or the final published versions. They should not be cited or distributed as the published version of this manuscript.

BMJ Open is an open access journal and the full, final, typeset and author-corrected version of record of the manuscript is available on our site with no access controls, subscription charges or pay-per-view fees (http://bmjopen.bmj.com).

If you have any questions on BMJ Open's open peer review process please email info.bmjopen@bmj.com

BMJ Open

Elements of Organization of Integrated Maternity Care and their Associations with Outcomes: A Scoping Review Protocol

Journal:	*BMJ Open*
Manuscript ID	bmjopen-2023-075344
Article Type:	Protocol
Date Submitted by the Author:	22-Jun-2023
Complete List of Authors:	Liebregts, Jolanda; Amsterdam UMC Locatie VUmc, Midwifery Science<br>Goodarzi , Bahareh ; Amsterdam UMC Locatie AMC, Midwifery Science<br>Valentijn, Pim; Maastricht University, Department of Health Services Research<br>Downe, Soo; University of Central Lancashire, research in childbirth and health<br>Erwich, Jan Jaap; University of Groningen, Obstetrics and Gynaecology<br>Burchell, George; Vrije Universiteit Amsterdam, Medical Library<br>Batenburg, Ronald; Netherlands Institute for Health Services Research,<br>de Vries, Eline; Centre for Nutrition, Prevention and Health Services, Department of Quality of Care and Health Economics; National Institute of Public Health and Primary Care, Leiden University Campus The Hague<br>de Jonge, Ank; AVAG, APH research institute, Amsterdam UMC, locatie VUMC, Midwifery Science<br>Verhoeven, Corine; Amsterdam UMC Locatie VUmc, Midwifery Science; University of Nottingham, Midwifery, School of Health Sciences<br>Study Group, VOICE; Amsterdam UMC Locatie VUmc Divisie 10, Midwifery Science
Keywords:	Organisation of health services < HEALTH SERVICES ADMINISTRATION & MANAGEMENT, OBSTETRICS, Patient-Centered Care, PERINATOLOGY, PUBLIC HEALTH

SCHOLARONE™
Manuscripts

Elements of Organization of Integrated Maternity Care and their Associations with Outcomes: A Scoping Review Protocol

J. Liebregts[1,2,3,4], B. Goodarzi[1,2,3,4], P. Valentijn[5,6], S. Downe[7], J.J.H.M. Erwich[8,], G.L. Burchell[10], R. Batenburg[11,12], E. de Vries[13,14], A. de Jonge[1,2,3,4], C.J. Verhoeven[1,2,4,9,15,16]

1. Amsterdam UMC location Vrije Universiteit Amsterdam, Midwifery Science, De Boelelaan 1117, Amsterdam, the Netherlands
2. Midwifery Academy Amsterdam Groningen, InHolland, Amsterdam, the Netherlands
3. Amsterdam Public Health, Quality of Care, Amsterdam, the Netherlands
4. The University of Groningen, University Medical Center Groningen, Department of General Practice & Elderly Care Medicine, PO Box 196, 9700 AD Groningen, the Netherlands
5. Essenburgh Research & Consultancy, Essenburgh Group, Harderwijk, Netherlands.
6. Department of Health Services Research, School for Public Health and Primary Care, Faculty of Health, Medicine and Life Sciences, Maastricht University, Maastricht, Netherlands.
7. The University of Central Lancashire, Preston, United Kingdom.
8. University of Groningen, University Medical Center Groningen, Department of Obstetrics & Gynecology, Groningen, The Netherlands
9. Amsterdam Reproduction and Development, Amsterdam, the Netherlands
10. Vrije Universiteit Amsterdam, Amsterdam, the Netherlands
11. Netherlands Institute for Health Services Research (NIVEL), 3513 CR Utrecht, The Netherlands.
12. Department of Sociology, Radboud University Nijmegen, 6525 XZ Nijmegen, The Netherlands.
13. Department of Quality of Care and Health Economics, Center for Nutrition, Prevention, and Health Services, National Institute of Public Health and the Environment (RIVM), Bilthoven, The Netherlands.
14. Department of Public Health and Primary Care, Leiden University Medical Center Campus The Hague, The Hague, The Netherlands.
15. Department of Obstetrics and Gynecology, Maxima Medical Centre, Veldhoven, Netherlands

For peer review only

16. Midwifery Department, School of Health Sciences, University of Nottingham, Nottingham, United Kingdom

Corresponding Author

J.D. Liebregts

De Boelelaan 1117, 1081 HV, Amsterdam, The Netherlands

Tel: +31 653861869

Email: j.d.liebregts@amsterdamumc.nl

Keywords

Organization of integrated care – Effectiveness - Health - Maternity care – Satisfaction

Word Count Protocol

2973 words

Word Count Abstract

292 words

For peer review only

Page 3 of 15 BMJ Open

ABSTRACT

The objective of this scoping review is to collate (a) the extent and type of evidence about elements of the organization of integrated maternity care and (b) their association with maternal and neonatal health, experiences of women and professionals, healthcare spending, and care processes.

Introduction Integrated care is considered as an enabling strategy in organising healthcare in terms of better quality, finances, personnel and sustainability. Developments in organization of maternity care follow this trend. The organization of care is a means to achieve the general aims and outcomes of healthcare systems. Organization itself is made up of a variety of smaller 'elements of organization'. Different elements of organization are implemented in different organizations and networks. We focus at 'What makes integrated maternity care work' in terms of circumstances and conditions that are enabling and the outcome-effects associated with these elements of organization.

Methods and analysis We conduct this review using guidelines of JBI and PRISMA-ScR (1, 2). We undertake a systematic search in the databases PubMed, Scopus, Cochrane, and PsychInfo. A machine learning tool, ASReview is used to select relevant papers (3). These papers are analyzed and classified thematically using analysis based on the framework of Valentijns' Rainbow model of integrated care (RMIC)(4, 5). The PCC framework for scoping reviews (1) is used. "Population" are elements of the organization of integrated maternity care. "Context" is high-income countries. "Concepts" are outcomes as stated in the objective of this review. We will include papers from 2012 onward, in Dutch or English language, which describe both "how the care is organized" (elements) and "outcomes" .

Ethics and dissemination Since this is a scoping review of previously published summary data, ethical approval for this study is not needed. Findings will be published in a peer-reviewed journal.

For peer review only - http://bmjopen.bmj.com/site/about/guidelines.xhtml

STRENGHTS AND LIMITATIONS OF THIS STUDY

- This scoping review will provide a comprehensive overview of elements of organization of integrated maternity care, their associations on outcomes and experiences and the level of integrated care they apply to.
- The study will ensure a thorough systematic procedure with the use of the Preferred Reporting Items for Systematic Reviews and Meta-Analyses Extension for Scoping Reviews (PRISMA-ScR).
- As the scoping review methodology limits our analysis to the information included in publications by the authors, we anticipate that some variables of interest will be under-reported and, thus, difficult to assess.
- The heterogeneity of organization and definition of integrated (maternity) care used in the existing literature, combined with the requirement of an English-language abstract, may limit our ability to locate all eligible studies.

For peer review only

Page 5 of 15 BMJ Open

INTRODUCTION

Many countries face challenges in organizing healthcare in terms of finances, personnel, and sustainability. Integrated care is considered as an important enabling strategy. Developments in maternity care follow this trend. The topic of integrated maternity care is complex and literature provides different definitions of integrated care. Based on their general perspective, we choose to use the definition of integrated maternity care by Allana et al (6); Integrated maternity care is “when the network(s) of multiple professionals and organizations across the maternity care and social care systems provide accessible, comprehensive and coordinated services to women who want to get pregnant, are pregnant and/or are up to 6 weeks after birth”. Organization of care is a means to obtain the general aims and outcomes of healthcare systems. The aims of healthcare are often described in frameworks like “Value-Based Healthcare”, “Triple Aim” and “Quadruple Aim”. In these frameworks, healthcare aims are defined as the improvement of population health, patient experiences, and enabling healthcare systems to be cost-efficient: a trio of goals commonly referred to as the ‘Triple Aim’ (7). In later publications, a fourth aim was added; the wellbeing and experiences of healthcare professionals (8). Along the same lines ‘Value-Based Health Care’ (VBHC) is to obtain the best outcomes for patients and populations with the optimal use of resources (money, time, carbon, and use of space) while adding value to the system (9). These frameworks are also referred to in the literature on integrated healthcare (7, 9, 10).
reality in modern-day maternity care is that it is an evolving complex field of interests, perspectives, and stakeholders like policymakers, financers, healthcare organizations, healthcare professionals, and clients. These different stakeholders all have different perspectives and expectations on the definition and aims of integrated (maternity) care (11) and little is known about how to organize integrated maternity care to meet these different aims. A closer look into the organization of integrated maternity care shows it is made up of a variety of smaller ‘elements of organization’. Elements such as standard inter professional consultation (MDO), risk selection model, group care, and bundled payment. In practice, there is a large variation in how integrated maternity care is organized. Different elements of the organization of care are implemented in different organizations and networks, while little is known about their effects (11). Furthermore, integrated care implies there are different levels of the organization; organization of care between client and professional (guidelines

For peer review only - http://bmjopen.bmj.com/site/about/guidelines.xhtml

and protocols), organization between professionals (who does what and when?), within healthcare organizations and healthcare organizations on a systemic level (payment system, legislation etcetera). To understand the complexity of integrated care organization and to guide empirical research, we will use the rainbow model of integrated care (RMIC) (6) to have a systematic understanding of the design, delivery, management, and evaluation of the organization of integrated care. We elaborate on 'What makes integrated maternity care work' not only by the associations of elements of organisation on the outcomes, but also in terms of circumstances and conditions under which integrated maternity care can contribute to the aims and outcomes of care. The aim of this scoping review is therefore to gain a better understanding of (a) the extent and type of evidence about elements of the organization of integrated maternity care, and (b) their association with the commonly accepted aims of integrated maternity care: maternal and neonatal health (7-9), experiences of women and professionals (8), healthcare spending (7, 9) and care processes (9). A preliminary search of MEDLINE, the Cochrane Database of Systematic Reviews, and JBI Evidence Synthesis was conducted on these aims and no current or underway systematic reviews or scoping reviews on the topic were identified.

REVIEW QUESTION

Review question: Which elements of the organization of integrated maternity care are associated with maternal and neonatal health outcomes, experiences of women and professionals, healthcare spending, and care processes?

KEYWORDS

Organization of integrated care – Effectiveness - Health - Maternity care – Satisfaction

ELIGIBILITY CRITERIA

In our scoping review, we elaborate on the Population - Context– Concept framework, recommended in the JBI manual for Scoping Reviews (1). In our review, "Population" does not consist of people but elements of the organization of integrated maternity care. The "Context" is defined as and limited to high-income countries while "Concepts" are the outcomes associated with the elements of organization of integrated maternity care.

For peer review only

Population Population consists of the various elements of the organization of integrated maternity care. We will explore the type and level of evidence found for these elements, their associations with outcomes as well as their distribution on the different levels of the RMIC framework.

Context The "Context" is limited to high-income countries because the organization of care in low and middle-income countries is very different. This implies that it would be meaningful to conduct a separate review in these settings.

Concept Concepts are maternal and neonatal outcomes (9), experiences of clients(12) and professionals (8), care delivery processes (13, 14), and healthcare spending (7, 8). We will include papers in which both elements of integrated maternity care and one or more of these outcomes are described.

Types of Sources This scoping review will consider both experimental and quasi-experimental study designs including systematic reviews, meta-analysis, randomized controlled trials, non-randomized controlled trials, before and after studies, and interrupted time-series studies. In addition, analytical observational studies including prospective and retrospective cohort studies, case-control studies, and analytical cross-sectional studies will be considered for inclusion. This review also considers descriptive observational study designs including case series and descriptive cross-sectional studies for inclusion. Qualitative studies including, but not limited to, designs such as phenomenology, grounded theory, ethnography, qualitative description, action research, and feminist research will be considered as well. Policy documents will also be considered for inclusion in this scoping review.

METHODS

The proposed scoping review will be conducted by the JBI methodology for scoping reviews (15) and the PRISMA extension for scoping reviews (PRISMA-ScR) (2). We analyse the literature using in- depth reading and analysing, in which we use Valentijn's Rainbow Model of Integrated Care (RMIC) as an organizing framework for the elements of the organization of integrated maternity care (5). The RMIC is chosen over other frameworks because it offers an open-ended, exploratory, broad view of integrated care that can be applied to various, diverse elements of the organization of maternity care. This ensures that consideration is given to different types of integration (clinical, professional, organizational, system, functional, and normative) at multiple levels (micro, meso, and macro) and varying degrees (linkage, coordination, full integration).

For peer review only

In our study we will cooperate with a team of experts involved in all levels of maternity care, to ensure a diverse perspective and discussion with the research team; professionals, delegates from national professional associations (i.e. client representatives, KNOV (midwives), NVOG (obstetricians), V&VN (nurses), KCKZ (maternity nurse), delegates from national associations of health insurance companies, policymakers (i.e. CPZ (college of perinatal care), professional education institutes, experts on organization science, maternity care, epidemiology, and economic analysis. The team of experts will be further referred to as the 'expert group'.

Search strategy

An initial limited search of PubMed was undertaken to identify articles on the topic. The text words contained in the titles and abstracts of relevant articles, and the index terms used to describe the articles were used to develop a full search strategy for PubMed, Scopus, PsychINFO, and Cochrane Library. We involve an information specialist, in consultation with the research team, to develop the final comprehensive literature search.

The search strategy, including all identified keywords and index terms, will be adapted for each included database and/or information source. The reference list of all included sources of evidence will be screened for additional studies.

Studies published in Dutch and English language and published from 2012 onward will be included as reviewers are fluent in both Dutch and English. We include papers from 2012 onward since this is the year the Member States of the WHO European Region endorsed the European health policy *Health 2020*, recognizing health system strengthening as one of four priority action areas and calls for people-centred health systems (11). This brought more attention to organization of integrated care.

The databases to be searched include PubMed, Scopus, PsychINFO, and Cochrane Library. Sources of unpublished studies/ gray literature to be searched include Google Scholar, key websites of interest (i.e. WHO, government agencies, and documents), hand searches of reference lists of papers and prior and additional knowledge of the expert team.

We focus on (a) elements of organization of integrated maternity care, (b) outcomes of maternal and neonatal health, women's and professionals' experiences, healthcare processes and healthcare costs and (c) levels of integrated care (RMIC). Potentially relevant references will

For peer review only

further be obtained from the retrieved publications and by contacting the expert team (snowball method).

Identified citations will be collected and uploaded into Endnote 20 (16) and duplicates will be removed. The in- and exclusion criteria will be applied based on a priori decisions made by the research team. Reviewers JL and BG will do the selection of relevant papers in two phases.

For the first selection of relevant papers, based on title and abstract, we will use an open-source machine learning-aided pipeline applying active learning: ASReview (3), version 1.1. This machine learning tool needs to be 'trained' to effectively rank the papers based on possible relevance. ASReview will be trained based on 'prior knowledge'; papers the research and expert team considers to be relevant to the topic at hand. In the active learning cycle, the model incrementally improves its predictions on the remaining unlabeled records, by which relevant records are identified as early in the process as possible.

To determine when to stop screening we elaborated on the stopping criterium for this screening, based on both knowledge within the research team and heuristic arguments as time and resources available. We decided to stop screening when a maximum of 400 papers are labeled relevant or 50 papers sequentially are labeled as irrelevant.

In the first stage, titles and abstracts will be screened by two independent reviewers (JL, BG) for assessment against the inclusion criteria for the review (Table 1). To ensure inter-reviewer agreement in both title and abstract screening as well as full-text screening, JL, and BG discuss uncertainties and specify and adjust the in- and exclusion criteria. Thereafter, in phase two, the full-text screening will be done by JL and BG. Any disagreements that arise between the reviewers at each stage of the selection process will be resolved through discussion, or with an additional reviewer/s (CV).

After we selected the relevant papers an export file (.csv) will be extracted out of ASReview and will be presented to the research team. In these files both the labeled papers (papers labeled as relevant or irrelevant) and unlabelled papers will be visible. They are put in order of relevance based on the machine learning tool.

When this first selection of relevant papers is finished, full texts and their citation details will be retrieved and imported into ASReview. The full text of selected papers will be assessed in detail against the inclusion and exclusion criteria (Table 1) by two independent reviewers (JL

For peer review only - http://bmjopen.bmj.com/site/about/guidelines.xhtml

and BG). Reasons for exclusion will be recorded and reported in the scoping review. The results of the search and the study inclusion process will be reported in full and presented in a Preferred Reporting Items for Systematic Reviews and Meta-analyses extension for scoping review (PRISMA-ScR) flow diagram (Figure 1) (2).

Relevant papers contain information on elements of the organization AND one or more of the outcomes of interest. Information about the organization and the associations with these outcomes will be analyzed.

Table 1. In and exclusion criteria for selecting relevant papers.

Inclusion	Exclusion
Elements of organization of integrated maternity care	Elements NOT related to the organization of care, i.e. content of care
Network(s) of multiple professionals and organizations across the maternity care and social care systems	
Information on accessible, comprehensive and coordinated services in maternity care	
Midwives, obstetricians/gynecologists, obstetric nurses/nurse midwives, maternity care assistants, and their clients/ patients (people who want to get pregnant, are pregnant and/or are up to 6 weeks after birth).	Articles in which either midwives or obstetricians play no role at all.
maternal and neonatal health; pregnancy outcomes, morbidity, mortality, and complications.	
patient experiences, healthcare professional experiences, well-being, satisfaction	
healthcare spending; costs, expenditure. -> money and assets -> I.e. more or fewer healthcare professionals/hours, etc	
Care processes; referral, intervention, transfer, medicalization.	
Peer-reviewed papers, articles in professional journals, policy documents, RCTs, Cohort and case-control studies, Case studies, Qualitative research, Editorials, Comments	abstracts of congresses and congress presentations, research protocols
Papers published between 2012 - 2022	Papers published < 2012
maternity care in high-income countries	Low and Middle-income countries<br>Healthcare is other than maternity care. Nonregular context -> refugees, war situation, pandemic.
Language: English and Dutch	Other languages than English and Dutch

For peer review only

For peer review only - http://bmjopen.bmj.com/site/about/guidelines.xhtml

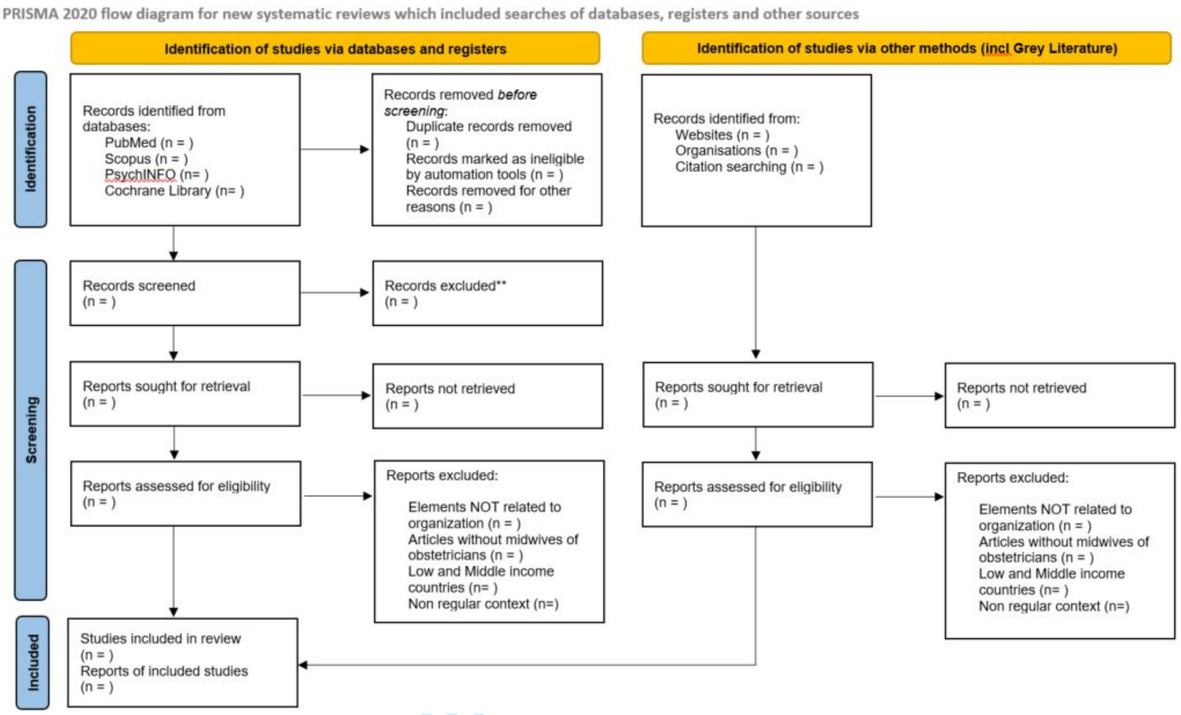

Figure 1. Flow diagram. PRISMA, Preferred Reporting Items for Systematic Reviews and Meta-Analysis (17)

Data Extraction

Data will be extracted from papers included in the scoping review by two independent reviewers (JL and BG) using a data extraction tool developed by the research team. The data extracted include specific details about (a) the elements of the organization of integrated maternity care in high-income countries, (b) specific details on associations of the elements of the organization on maternal and neonatal health, experiences of clients and healthcare professionals, care processes, and healthcare spending, and (c) the taxonomy provided by the RMIC will be used to extract data on different levels of integrated care according to the RMIC framework (18). Also, data will be categorized based on the methods used in the included papers to analyse the level of evidence per element of organization.

These data provide information on the extent and type of evidence available on elements of integrated maternity care on their associations with outcomes and the different levels of integrated care.

For the data extraction we use a method based on the framework analysis (4) using the framework of RMIC to identify the different elements of organization of integrated maternity care, their associations with outcomes and the level of integrated care they belong to.

For peer review only

For peer review only - http://bmjopen.bmj.com/site/about/guidelines.xhtml

A draft extraction form is provided (see Supplementary Table 1). The draft data extraction tool will be modified and revised as necessary during the process of extracting data from each included evidence source. Modifications will be detailed in the scoping review.

We will follow the same procedure as previously described in the literature selection. The research team determines the criteria for the data extraction table (Supplementary Table 1). Two reviewers (JL and BG) will extract data independently out of five randomly selected included papers and discuss until they reach a complete agreement. Any disagreements that arise between the reviewers JL and BG will be resolved through discussion, or with an additional reviewer (CV). Full data extraction will be started after reviewers have obtained sufficient agreement. If appropriate, authors of papers will be contacted to request missing or additional data, where required.

Data Analysis and Presentation

Data will be presented initially in a regular table based on the data extraction form (Supplementary Table 1) Depending on the nature of emerging associations, we plan to use a range of graphical solutions to illustrate the findings.

For peer review only

Page 13 of 15 BMJ Open

ACKNOWLEDGEMENTS

Patient and Public involvement

None

Authors Contributions

Contributors JL, BG, PV, JJE, GB, RB, EdV, SD, AdJ and CV contributed to the development of this study protocol. JL conceptualized the research question, designed the study and prepared the first draft of the study protocol. BG and SD provided methodological expertise. PV provided expertise on the Rainbow Model of Integrated Care. RB and EdV provide expertise in funding, research and organization of integrated maternity care. JJE, CV and AdJ provided methodological expertise, expertise from their previous research on the topic of organization of integrated maternity care. GB is librarian at AmsterdamUMC and contributes his expertise in the systematic search and reporting according PRISMA-ScR. All authors have contributed to the study design and revising the protocol. All authors have approved the final manuscript. All authors agree to be accountable for all aspects of the work in ensuring that questions related to the accuracy or integrity of any part of the work are appropriately investigated and resolved

Funding

This study is funded by ZonMW: The Dutch association for health research and development, program 'Zwangerschap en geboorte II', grant number 05430052110002

Competing Interests

None declared

Patient and Public involvement

Patients and/or the public were not involved in the design, or conduct, or reporting, or dissemination plans of this research

Further Acknowledgements

For peer review only - http://bmjopen.bmj.com/site/about/guidelines.xhtml

This review is part of a Ph.D. trajectory of Liebregts, J.D.

This scoping review is part of the Dutch VOICE study – Variations in Organization of Integrated CarE.

REFERENCES

1. Aromataris E MZ. JBI Manual for Evidence Synthesis. 2020 [Available from: https://synthesismanual.jbi.global.
2. Tricco AC, Lillie E, Zarin W, O'Brien KK, Colquhoun H, Levac D, et al. PRISMA Extension for Scoping Reviews (PRISMA-ScR): Checklist and Explanation. Ann Intern Med. 2018;169(7):467-73.
3. van de Schoot R, de Bruin J, Schram R, Zahedi P, de Boer J, Weijdema F, et al. An open source machine learning framework for efficient and transparent systematic reviews. Nature Machine Intelligence. 2021;3(2):125-33.
4. Booth A, Carroll C. How to build up the actionable knowledge base: the role of 'best fit' framework synthesis for studies of improvement in healthcare. BMJ Qual Saf. 2015;24(11):700-8.
5. Valentijn PP, Schepman SM, Opheij W, Bruijnzeels MA. Understanding integrated care: a comprehensive conceptual framework based on the integrative functions of primary care. Int J Integr Care. 2013;13:e010.
6. Allana A, Tavares W, Pinto AD, Kuluski K. Designing and Governing Responsive Local Care Systems - Insights from a Scoping Review of Paramedics in Integrated Models of Care. Int J Integr Care. 2022;22(2):5.
7. Berwick DM, Nolan TW, Whittington J. The triple aim: care, health, and cost. Health Aff (Millwood). 2008;27(3):759-69.
8. Bodenheimer T, Sinsky C. From triple to quadruple aim: care of the patient requires care of the provider. Ann Fam Med. 2014;12(6):573-6.
9. De Jonge A, Downe S, Page L, Devane D, Lindgren H, Klinkert J, et al. Value based maternal and newborn care requires alignment of adequate resources with high value activities. BMC Pregnancy Childbirth. 2019;19(1):428.
10. Kokko P. Improving the value of healthcare systems using the Triple Aim framework: A systematic literature review. Health Policy. 2022;126(4):302-9.
11. Organization WH. WHO Integrated care models overview. 2016.
12. Downe S, Finlayson K, Oladapo OT, Bonet M, Gülmezoglu AM. What matters to women during childbirth: A systematic qualitative review. PLoS ONE. 2018;13(4):e0194906.
13. Renfrew MJ, McFadden A, Bastos MH, Campbell J, Channon AA, Cheung NF, et al. Midwifery and quality care: findings from a new evidence-informed framework for maternal and newborn care. Lancet. 2014;384(9948):1129-45.
14. Miller S, Abalos E, Chamillard M, Ciapponi A, Colaci D, Comandé D, et al. Beyond too little, too late and too much, too soon: a pathway towards evidence-based, respectful maternity care worldwide. Lancet. 2016;388(10056):2176-92.
15. Peters MDJ GC, McInerney P, Munn Z, Tricco AC, Khalil, H. . Chapter 11: Scoping Reviews (2020 version). In: Aromataris E, Munn Z (Editors). JBI Manual for Evidence Synthesis, JBI, 2020 2020 [Available from: https://synthesismanual.jbi.global.
16. Team TE. Endnote. EndNote 20 ed. Philadelphia, PA: Clarivate; 2013.
17. Page MJ, McKenzie JE, Bossuyt PM, Boutron I, Hoffmann TC, Mulrow CD, et al. The PRISMA 2020 statement: an updated guideline for reporting systematic reviews. Bmj. 2021;372:n71.
18. Valentijn PP, Boesveld IC, van der Klauw DM, Ruwaard D, Struijs JN, Molema JJ, et al. Towards a taxonomy for integrated care: a mixed-methods study. Int J Integr Care. 2015;15:e003.

For peer review only

For peer review only

For peer review only - http://bmjopen.bmj.com/site/about/guidelines.xhtml

PRISMA 2020 flow diagram for new systematic reviews which included searches of databases, registers and other sources

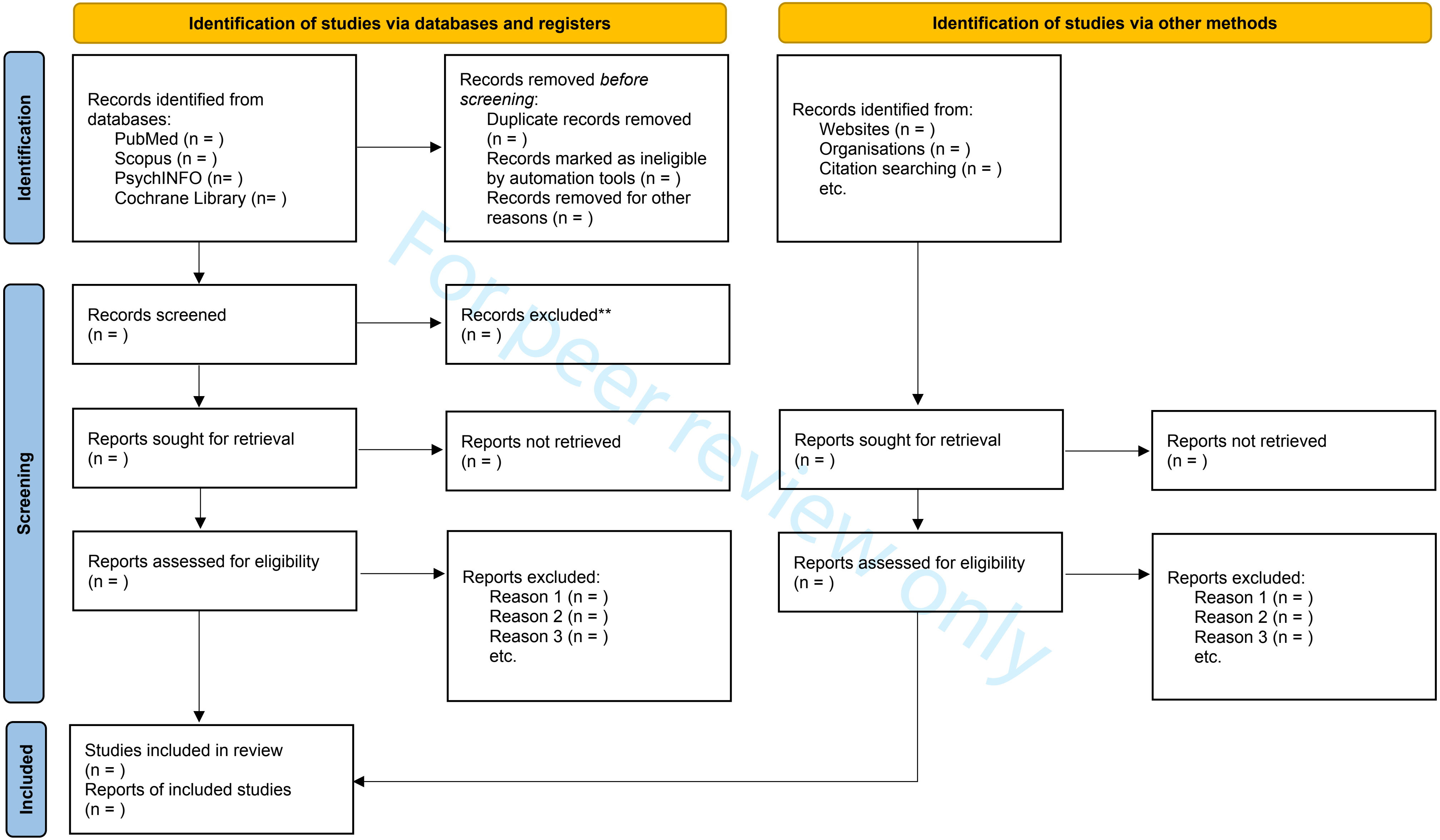

*Consider, if feasible to do so, reporting the number of records identified from each database or register searched (rather than the total number across all databases/registers).
**If automation tools were used, indicate how many records were excluded by a human and how many were excluded by automation tools.

From: Page MJ, McKenzie JE, Bossuyt PM, Boutron I, Hoffmann TC, Mulrow CD, et al. The PRISMA 2020 statement: an updated guideline for reporting systematic reviews. BMJ 2021;372:n71. doi: 10.1136/bmj.n71. For more information, visit: http://www.prisma-statement.org/

For peer review only

For peer review only - http://bmjopen.bmj.com/site/about/guidelines.xhtml

Supplementary Table 1. Draft Data Extraction Table.

Element of integrated maternity care	Outcomes					Levels RMIC								Level of evidence
Element of integrated maternity care	Outcome 1. Maternal and neonatal health	Outcome 2. Experiences of professionals	Outcome 3. Experiences of mothers	Outcome 4. Healthcare processes	Healthcare spending	Vertical – Horizontal organization:	Degree of integration	Clinical integration	Professional integration	Organizational integration:	System Integration	Functional enablers:	Normative enablers:	Level of evidence
e.g. mothercouncil														
e.g. multidisciplinary consultation														

For peer review only

BMJ Open

Elements of Organization of Integrated Maternity Care and their Associations with Outcomes: A Scoping Review Protocol

Journal:	*BMJ Open*
Manuscript ID	bmjopen-2023-075344.R1
Article Type:	Protocol
Date Submitted by the Author:	22-Nov-2023
Complete List of Authors:	Liebregts, Jolanda; Amsterdam UMC Locatie VUmc, Midwifery Science<br>Goodarzi , Bahareh ; Amsterdam UMC Locatie AMC, Midwifery Science<br>Valentijn, Pim; Maastricht University, Department of Health Services Research<br>Downe, Soo; University of Central Lancashire, research in childbirth and health<br>Erwich, Jan Jaap; University of Groningen, Obstetrics and Gynaecology<br>Burchell, George; Vrije Universiteit Amsterdam, Medical Library<br>Batenburg, Ronald; Netherlands Institute for Health Services Research,<br>de Vries, Eline; Centre for Nutrition, Prevention and Health Services, Department of Quality of Care and Health Economics; National Institute of Public Health and Primary Care, Leiden University Campus The Hague<br>de Jonge, Ank; AVAG, APH research institute, Amsterdam UMC, locatie VUMC, Midwifery Science<br>Verhoeven, Corine; Amsterdam UMC Locatie VUmc, Midwifery Science; University of Nottingham, Midwifery, School of Health Sciences<br>Study Group, VOICE; Amsterdam UMC Locatie VUmc Divisie 10, Midwifery Science
**Primary Subject Heading**:	Obstetrics and gynaecology
Secondary Subject Heading:	Public health, Evidence based practice, Health policy, Patient-centred medicine
Keywords:	Organisation of health services < HEALTH SERVICES ADMINISTRATION & MANAGEMENT, OBSTETRICS, Patient-Centered Care, PERINATOLOGY, PUBLIC HEALTH

SCHOLARONE™
Manuscripts

Elements of Organization of Integrated Maternity Care and their Associations with Outcomes: A Scoping Review Protocol

J. Liebregts[1,2,3,4], B. Goodarzi[1,2,3,4], P. Valentijn[5,6], S. Downe[7], J.J.H.M. Erwich[8,], G.L. Burchell[10], R. Batenburg[11,12], E. de Vries[13,14], A. de Jonge[1,2,3,4], C.J. Verhoeven[1,2,4,9,15,16], VOICE study group[1]

1. Amsterdam UMC location Vrije Universiteit Amsterdam, Midwifery Science, De Boelelaan 1117, Amsterdam, the Netherlands
2. Midwifery Academy Amsterdam Groningen, InHolland, Amsterdam, the Netherlands
3. Amsterdam Public Health, Quality of Care, Amsterdam, the Netherlands
4. The University of Groningen, University Medical Center Groningen, Department of General Practice & Elderly Care Medicine, PO Box 196, 9700 AD Groningen, the Netherlands
5. Essenburgh Research & Consultancy, Essenburgh Group, Harderwijk, Netherlands.
6. Department of Health Services Research, School for Public Health and Primary Care, Faculty of Health, Medicine and Life Sciences, Maastricht University, Maastricht, Netherlands.
7. The University of Central Lancashire, Preston, United Kingdom.
8. University of Groningen, University Medical Center Groningen, Department of Obstetrics & Gynecology, Groningen, The Netherlands
9. Amsterdam Reproduction and Development, Amsterdam, the Netherlands
10. Vrije Universiteit Amsterdam, Amsterdam, the Netherlands
11. Netherlands Institute for Health Services Research (NIVEL), 3513 CR Utrecht, The Netherlands.
12. Department of Sociology, Radboud University Nijmegen, 6525 XZ Nijmegen, The Netherlands.
13. Department of Quality of Care and Health Economics, Center for Nutrition, Prevention, and Health Services, National Institute of Public Health and the Environment (RIVM), Bilthoven, The Netherlands.
14. Department of Public Health and Primary Care, Leiden University Medical Center Campus The Hague, The Hague, The Netherlands.
15. Department of Obstetrics and Gynecology, Maxima Medical Centre, Veldhoven, Netherlands

For peer review only

16. Midwifery Department, School of Health Sciences, University of Nottingham, Nottingham, United Kingdom

Corresponding Author

J.D. Liebregts

De Boelelaan 1117, 1081 HV, Amsterdam, The Netherlands

Tel: +31 653861869

Email: j.d.liebregts@amsterdamumc.nl

Keywords

Organization of integrated care – Effectiveness - Health - Maternity care – Satisfaction

Word Count Protocol

2538 words

Word Count Abstract

291 words

For peer review only

Page 3 of 15 BMJ Open

ABSTRACT

Introduction Integrated care is seen as an enabling strategy in organising healthcare to improve quality, finances, personnel and sustainability. Developments in organization of maternity care follow this trend. The way care is organised should support the general aims and outcomes of healthcare systems. Organization itself consists of a variety of smaller 'elements of organization'. Various elements of organization are implemented in different organizations and networks. We will examine which elements of integrated maternity care are associated with maternal and neonatal health outcomes, experiences of women and professionals, healthcare spending and care processes.

Methods and analysis We will conduct this review using the JBI methodology for scoping reviews and the reporting guideline PRISMA-ScR. We will undertake a systematic search in the databases PubMed, Scopus, Cochrane, and PsychInfo. A machine learning tool, ASReview will be used to select relevant papers. These papers will be analysed and classified thematically using the framework of the Rainbow Model of Integrated Care (RMIC). The Population Concept Context framework for scoping reviews will be used in which "Population" is defined as elements of the organization of integrated maternity care, "Context" as high-income countries and "Concepts" as outcomes stated in the objective of this review. We will include papers from 2012 onward, in Dutch or English language, which describe both "how the care is organized" (elements) and "outcomes".

Ethics and dissemination Since this is a scoping review of previously published summary data, ethical approval for this study is not needed. Findings will be published in a peer-reviewed international journal, discussed in a webinar and presented at (inter)national conferences and meetings of professional associations.

The findings of this scoping review will give insight into the nature and effectiveness of elements of integrated care and will generate hypotheses for further research.

For peer review only

For peer review only - http://bmjopen.bmj.com/site/about/guidelines.xhtml

STRENGHTS AND LIMITATIONS OF THIS STUDY

- We will use an AI machine learning tool, ASReview, which will allow us to efficiently select the relevant papers from a large amount of literature found in the systematic search.
- The Rainbow Model for Integrated Care will be used as an explanatory framework to see what evidence is available on which level of integrated care and subsequently which information is missing.
- The members of the research team and expert group working on this scoping review represent different views in maternity care, this ensures a broad scope and reduction of bias.
- We will focus on organization of integrated maternity care in high income countries and exclude literature from low and middle income countries; this a limitation for drawing general, global conclusions.

For peer review only

INTRODUCTION

Many countries face challenges in organizing healthcare in terms of finances, personnel, and sustainability. Integrating different echelons of care is seen as an enabling strategy in this. Developments in maternity care follow this trend. The topic of integrated maternity care is complex and the literature shows different definitions of integrated care. We adapted the definition from Allana et al (1) to integrated maternity care; "when the network(s) of multiple professionals and organizations across the maternity care and social care systems provide accessible, comprehensive and coordinated services to women who want to get pregnant, are pregnant and/or are up to 6 weeks after birth". The way integrated maternity care is organised should support the general aims and outcomes of healthcare systems. Generally accepted goals of healthcare systems are: improving population health, enhancing client experiences and reducing costs per person (Triple Aim) (2, 3). Improving healthcare professionals experiences has subsequently been added as well (Quadruple Aim)(4) Healthcare goals can also be formulated in terms of sustainability, that is seeking the best outcomes for clients and the population against optimal use of resources (time, carbon, use of space) (Value Based Healthcare)(5) The organisation of integrated maternity care is a means to achieve these goals. However, due to different perspectives, cultures and interests of stakeholders organising care is complex. The organisation itself consists of a variety of smaller 'organisational elements', including different models of risk selection, group-based antenatal care, continuity of care and different payment systems. Different elements of the organisation of care are implemented in different organisations and networks, while little is known about their effects effects (6). Furthermore, integrated care concerns different levels of the organization; organization at client level (guidelines and protocols), between professionals (who does what and when?), within healthcare organizations and healthcare organizations on a systemic level (payment system, legislation etcetera). We will use the Rainbow Model of Integrated Care (RMIC) to obtain a systemic understanding of the design, delivery, management and evaluation of the organisation of integrated care (7). We will examine 'What makes integrated maternity care work' not only by identifying elements of organisation of integrated care and their associations with outcomes,

For peer review only - http://bmjopen.bmj.com/site/about/guidelines.xhtml

but also by mapping the circumstances and conditions under which integrated maternity care can contribute to the aims and outcomes of care. The aim of this scoping review is therefore to gain a better understanding of the amount and type of evidence on (a) elements of the organization of integrated maternity care, and (b) their association with maternal and neonatal health (2, 5, 8), experiences of women and professionals (8), healthcare spending (2, 5) and care processes (5). A preliminary search of MEDLINE, the Cochrane Database of Systematic Reviews, and JBI Evidence Synthesis was conducted and no completed of ongoing systematic reviews or scoping reviews on the topic were identified.

REVIEW QUESTION

Review question: Which elements of the organization of integrated maternity care are associated with maternal and neonatal health outcomes, experiences of women and professionals, healthcare spending, and care processes?

KEYWORDS

Organization of integrated care – Effectiveness - Health - Maternity care – Satisfaction

ELIGIBILITY CRITERIA

In our scoping review, we will use the Population - Context– Concept framework, recommended in the JBI manual for Scoping Reviews (9);

Population The “Population” in this review consists of the various elements of the organization of integrated maternity care. We will explore the type and level of evidence found for these elements, their associations with outcomes as well as their distribution on the different levels of the RMIC framework.

Context The “Context” is limited to high-income countries because this study is designed to inform practice in high income countries. In addition, the organization of care in low and

For peer review only - http://bmjopen.bmj.com/site/about/guidelines.xhtml

middle-income countries is very different. This implies that it would be meaningful to conduct a separate review in these settings.

Concept The "Concepts" in this review are maternal and neonatal outcomes (5), experiences of clients (10) and professionals (8), care delivery processes (11, 12), and healthcare spending (2, 8).

Types of Studies This scoping review will consider both experimental and quasi-experimental study designs including systematic reviews, meta-analysis, randomized controlled trials, non-randomized controlled trials, before and after studies, and interrupted time-series studies. In addition, analytical observational studies including prospective and retrospective cohort studies, case-control studies, and cross-sectional studies will be considered for inclusion. We will also consider descriptive observational study designs for inclusion such as case series and cross-sectional studies. Qualitative studies based on, but not limited to, phenomenology, grounded theory, ethnography, action research, and feminist research will be considered as well. In addition policy documents will be considered.

METHODS

The proposed scoping review will be conducted using the JBI methodology for scoping reviews (13) and the reporting guideline PRISMA, extension for scoping reviews (see Supplementary File 1 for the Prisma ScR Checklist) (14). We will analyse the literature using in-depth reading and analysis, in which we will use Valentijn's Rainbow Model of Integrated Care (RMIC) as an organizing framework for the elements of the organization of integrated maternity care (7). The RMIC is chosen over other frameworks because it offers an open-ended, exploratory, broad view of integrated care that can be applied to various, diverse elements of the organization of maternity care. This will ensure that consideration will be given to different types of integration (clinical, professional, organizational, system, functional, and normative) at multiple levels (micro, meso, and macro) and varying degrees (linkage, coordination, full integration).

In our study the research team will cooperate with a team of experts, members of the VOICE study group, who are involved in different levels of maternity care who will advise on all aspects of the study. To ensure a diversity of perspectives within the expert group the following representatives will be included: professionals, representatives from national professional

For peer review only - http://bmjopen.bmj.com/site/about/guidelines.xhtml

associations (i.e. client representatives, the Dutch Organization of Midwives (KNOV), the Dutch Society of Obstetricians (NVOG),the Dutch association of nurses and carers (V&VN), Knowledge Centre Maternity Care (KCKZ), representatives from health insurance companies, policymakers (i.e. Dutch College of Perinatal Care (CPZ), professional education institutes and experts on organization science, maternity care, epidemiology, and economic analysis. The team of experts will be further referred to as the 'expert group'.

Search strategy

An initial limited search of PubMed was undertaken to identify articles on the topic. The text of titles and abstracts of relevant articles and the key words used to describe the articles will be used to develop a full search strategy for PubMed, Scopus, PsychINFO, and Cochrane Library with the help of an information specialist (see Supplementary File 2). We will consult the expert group to develop the final comprehensive literature search.

The search strategy, including all identified keywords and index terms, will be adapted for each included database and/or information source by the information specialist. The reference list of all included sources of evidence will be screened for additional studies.

Studies published in Dutch and English language will be included as reviewers are fluent in both Dutch and English. We will include papers from 2012 onward since this is the year the Member States of the WHO European Region endorsed the European health policy *Health 2020*, acknowledging health system strengthening as one of four priority action areas and calls for people-centred health systems (6). This generated attention to the organization of integrated care.

The databases to be searched include PubMed, Scopus, PsychINFO, and Cochrane Library. Sources of unpublished studies and grey literature to be searched include Google Scholar, key websites of interest (i.e. WHO, government agencies), hand searches of reference lists of papers and prior and additional knowledge of the expert group .

We will focus on (a) elements of organization of integrated maternity care, (b) outcomes of maternal and neonatal health, women's and professionals' experiences, healthcare processes and healthcare costs and (c) levels of integrated care (RMIC). Potentially relevant references will further be obtained from the retrieved publications and by consulting the expert group (snowball method).

For peer review only - http://bmjopen.bmj.com/site/about/guidelines.xhtml

Identified citations will be collected and uploaded into Endnote 20 (15) and duplicates will be removed. The in- and exclusion criteria will be applied based on a priori decisions made by the research team. Reviewers JL and BG will do the selection of relevant papers in two phases.

For the first selection of relevant papers, based on title and abstract, we will use an open-source machine learning-aided pipeline applying active learning: ASReview (16), version 1.1. This machine learning tool needs to be 'trained' to effectively rank the papers based on possible relevance. ASReview will be trained based on 'prior knowledge'; papers the research team and expert group considers to be relevant to the topic at hand. In the active learning cycle, the model incrementally improves its predictions on the remaining unlabelled records, by which relevant records are identified as early in the process as possible.

To determine when to stop screening we elaborated on the stopping criterion for this screening, based on both knowledge within the research team and heuristic arguments as time and resources available. We will stop screening when a maximum of 400 papers are labelled relevant or 50 papers sequentially are labelled as irrelevant.

In the first stage, titles and abstracts will be screened by two independent reviewers (JL, BG) for assessment against the inclusion criteria for the review (Table 1). To ensure inter-reviewer agreement in both title and abstract screening as well as full-text screening, JL, and BG discuss uncertainties and specify and adjust the in- and exclusion criteria. In phase two, the full-text screening will be done by JL and BG. Any disagreements that arise between the reviewers at each stage of the selection process will be resolved through discussion, or with a third reviewer (CV).

After we selected the relevant papers an export file (.csv) will be extracted out of ASReview and will be presented to the research team and expert group. In these files both the labelled papers (papers labelled as relevant or irrelevant) and unlabelled papers will be visible. They are put in order of relevance based on the machine learning tool.

When this first selection of relevant papers is finished, full texts and their citation details will be retrieved and imported into ASReview. The full text of selected papers will be assessed in detail against the inclusion and exclusion criteria by two independent reviewers (JL and BG) (Table 1). Reasons for exclusion will be recorded and reported in the scoping review. The results of the search and the study inclusion process will be reported in full and presented in

For peer review only - http://bmjopen.bmj.com/site/about/guidelines.xhtml

line with the Preferred Reporting Items for Systematic Reviews and Meta-analyses extension for scoping review (PRISMA-ScR) flow diagram (Figure 1) (14).

Relevant papers will contain information on elements of the organization AND one or more of the outcomes of interest.

Table 1. In and exclusion criteria for selecting relevant papers.

**Inclusion**	**Exclusion**
Elements of organization of integrated maternity care	Elements NOT related to the organization of care, i.e. content of care
Network(s) of multiple professionals and organizations across the maternity care and social care systems	
Information on accessible, comprehensive and coordinated services in maternity care	
Midwives, obstetricians obstetric nurses/nurse midwives, maternity care assistants, and their clients/ patients (people who want to get pregnant, are pregnant and/or are up to 6 weeks after birth).	Articles in which either midwives or obstetricians play no role at all.
Maternal and neonatal health; pregnancy outcomes, morbidity, mortality, and complications.	
Patient experiences, healthcare professional experiences, well-being, satisfaction	
Healthcare spending; costs, expenditure. -> money and assets -> I.e. more or fewer healthcare professionals/hours, etc	
Care processes; referral, intervention, transfer, medicalization.	
Peer-reviewed papers, articles in professional journals, policy documents, RCTs, Cohort and case-control studies, Case studies, Qualitative research, Editorials, Comments	Abstracts of congresses and congress presentations, research protocols
Papers published between 2012 - 2022	Papers published before 2012
Maternity care in high-income countries	Low and Middle-income countries<br>Healthcare is other than maternity care. Nonregular context -> refugees, war situation, pandemic.
Language: English and Dutch	Other languages than English and Dutch

For peer review only - http://bmjopen.bmj.com/site/about/guidelines.xhtml

Figure 1. Flow diagram of the study (17)

Data Extraction

Data will be extracted from papers included in the scoping review by two independent reviewers (JL and BG) using a data extraction tool developed by the research team. The data extracted include specific details about (a) the elements of the organization of integrated maternity care in high-income countries, (b) specific details on associations of the elements of the organization on maternal and neonatal health, experiences of clients and healthcare professionals, care processes, and healthcare spending, and (c) the taxonomy provided by the RMIC will be used to extract data on different levels of integrated care according to the RMIC framework (18). Also, data will be categorized based on the methods used in the included papers to analyse the level of evidence per element of organization.

For the data extraction we will use a method based on the framework analysis (19) using the framework of RMIC to identify the different elements of organization of integrated maternity care, their associations with outcomes and the level of integrated care they belong to.

A draft extraction form is provided in Supplementary Table 1. The draft data extraction tool will be modified and revised as necessary during the process of extracting data from each included evidence source. Modifications will be detailed in the article with the results of the scoping review

We will follow the same procedure as previously described in the literature selection. The research team and expert group determine the criteria for the data extraction table (Supplementary Table 1). Two reviewers (JL and BG) will extract data independently out of three times twenty randomly selected included papers and discuss until they reach a complete agreement. Any disagreements that arise between the reviewers JL and BG will be resolved through discussion, or with a third reviewer (CV). Full data extraction will only be started after reviewers have obtained sufficient agreement. If appropriate, authors of papers will be contacted to request missing or additional data, where required.

Data Analysis and Presentation

Data will be presented initially in a regular table based on the data extraction form (Table 1) Depending on the nature of emerging associations, we plan to use a range of graphical solutions to illustrate the findings.

For peer review only - http://bmjopen.bmj.com/site/about/guidelines.xhtml

Patient and Public involvement

Patients and/or the public were not involved in the design, or conduct, or reporting, or dissemination plans of this research.

Ethics and dissemination

This is a scoping review of previously published summary data. The Amsterdam UMC medical ethical review committee confirmed that ethical approval for this study is not needed (METC number 2022.053). Findings will be published in a peer-reviewed international journal, discussed in a Dutch national level webinar for professionals, scientists and policymakers and presented at (inter)national conferences and meetings of professional associations.

For peer review only

ACKNOWLEDGEMENTS

Collaborators

In addition to the authors of this scoping review, the VOICE study group comprises; Irene de Graaf, Puck van Heemstra, Hester Rippen, Jeroen Struijs, Susanne Zuidhof, Inge Boesveld, Anouk Kaiser, Mirjam Fransen, Durk Berks, Pauline Haga, Katarzyna Burzynska, Joland Vermolen, Lucienne Bakker, Mariëtte Hoogsteder, Lillian Peters, Conny Vreugdehil, Suzanne Thompson, Aimée Sparendam-Bruijnincx, Betty de Vries, Elle Struijf, Lillianne van der Velde, Bert Horlings, Marianne Nieuwenhuijze, Marc Roosenboom and Simone Plaizier.

Authors Contributions

Contributors JL, BG, PV, JJE, GB, RB, EdV, SD, AdJ and CV contributed to the development of this study protocol. JL, AdJ and CV conceptualized the research question, designed the study and prepared the first draft of the study protocol. BG,SD JJE, AdJ and CV provided methodological expertise. PV provided expertise on the Rainbow Model of Integrated Care. RB and EdV provide expertise in funding, research and organization of integrated maternity care. GB is an information specialist at AmsterdamUMC and contributes his expertise in the systematic search and reporting according PRISMA-ScR. All authors have contributed to the study design and revising the protocol. All authors have approved the final manuscript. All authors agree to be accountable for all aspects of the work in ensuring that questions related to the accuracy or integrity of any part of the work are appropriately investigated and resolved. The VOICE study group was part of the conceptualisation of the VOICE study grant proposal.

Funding

This scoping review is part of the Dutch VOICE study – Variations in Organization of Integrated CarE, which is funded by ZonMW,the Dutch association for health research and development, program ‘Zwangerschap en geboorte II’, grant number 05430052110002

Competing Interests

For peer review only

None declared

Further Acknowledgements

This review is part of a Ph.D. trajectory of Liebregts, J.D.

REFERENCES

1. Allana A, Tavares W, Pinto AD, Kuluski K. Designing and Governing Responsive Local Care Systems - Insights from a Scoping Review of Paramedics in Integrated Models of Care. Int J Integr Care. 2022;22(2):5.
2. Berwick DM, Nolan TW, Whittington J. The triple aim: care, health, and cost. Health Aff (Millwood). 2008;27(3):759-69.
3. Kokko P. Improving the value of healthcare systems using the Triple Aim framework: A systematic literature review. Health Policy. 2022;126(4):302-9.
4. An W. Capsule Commentary on Mandl et al., Provider Collaboration: Cohesion, Constellations, and Shared Patients. Journal of General Internal Medicine. 2014;29(11):1530.
5. De Jonge A, Downe S, Page L, Devane D, Lindgren H, Klinkert J, et al. Value based maternal and newborn care requires alignment of adequate resources with high value activities. BMC Pregnancy Childbirth. 2019;19(1):428.
6. Organization WH. WHO Integrated care models overview. 2016.
7. Valentijn PP, Schepman SM, Opheij W, Bruijnzeels MA. Understanding integrated care: a comprehensive conceptual framework based on the integrative functions of primary care. Int J Integr Care. 2013;13:e010.
8. Bodenheimer T, Sinsky C. From triple to quadruple aim: care of the patient requires care of the provider. Ann Fam Med. 2014;12(6):573-6.
9. Aromataris E MZ. JBI Manual for Evidence Synthesis. 2020 [Available from: https://synthesismanual.jbi.global.
10. Downe S, Finlayson K, Oladapo OT, Bonet M, Gülmezoglu AM. What matters to women during childbirth: A systematic qualitative review. PLoS ONE. 2018;13(4):e0194906.
11. Renfrew MJ, McFadden A, Bastos MH, Campbell J, Channon AA, Cheung NF, et al. Midwifery and quality care: findings from a new evidence-informed framework for maternal and newborn care. Lancet. 2014;384(9948):1129-45.
12. Miller S, Abalos E, Chamillard M, Ciapponi A, Colaci D, Comandé D, et al. Beyond too little, too late and too much, too soon: a pathway towards evidence-based, respectful maternity care worldwide. Lancet. 2016;388(10056):2176-92.
13. Peters MDJ GC, McInerney P, Munn Z, Tricco AC, Khalil, H. . Chapter 11: Scoping Reviews (2020 version). In: Aromataris E, Munn Z (Editors). JBI Manual for Evidence Synthesis, JBI, 2020 2020 [Available from: https://synthesismanual.jbi.global.
14. Tricco AC, Lillie E, Zarin W, O'Brien KK, Colquhoun H, Levac D, et al. PRISMA Extension for Scoping Reviews (PRISMA-ScR): Checklist and Explanation. Ann Intern Med. 2018;169(7):467-73.
15. Team TE. Endnote. EndNote 20 ed. Philadelphia, PA: Clarivate; 2013.
16. van de Schoot R, de Bruin J, Schram R, Zahedi P, de Boer J, Weijdema F, et al. An open source machine learning framework for efficient and transparent systematic reviews. Nature Machine Intelligence. 2021;3(2):125-33.

For peer review only - http://bmjopen.bmj.com/site/about/guidelines.xhtml

Page **15** of **15** **BMJ Open**

17. Page MJ, McKenzie JE, Bossuyt PM, Boutron I, Hoffmann TC, Mulrow CD, et al. The PRISMA 2020 statement: an updated guideline for reporting systematic reviews. Bmj. 2021;372:n71.
18. Valentijn PP, Boesveld IC, van der Klauw DM, Ruwaard D, Struijs JN, Molema JJ, et al. Towards a taxonomy for integrated care: a mixed-methods study. Int J Integr Care. 2015;15:e003.
19. Booth A, Carroll C. How to build up the actionable knowledge base: the role of 'best fit' framework synthesis for studies of improvement in healthcare. BMJ Qual Saf. 2015;24(11):700-8.

For peer review only

PRISMA 2020 flow diagram for new systematic reviews which included searches of databases, registers and other sources

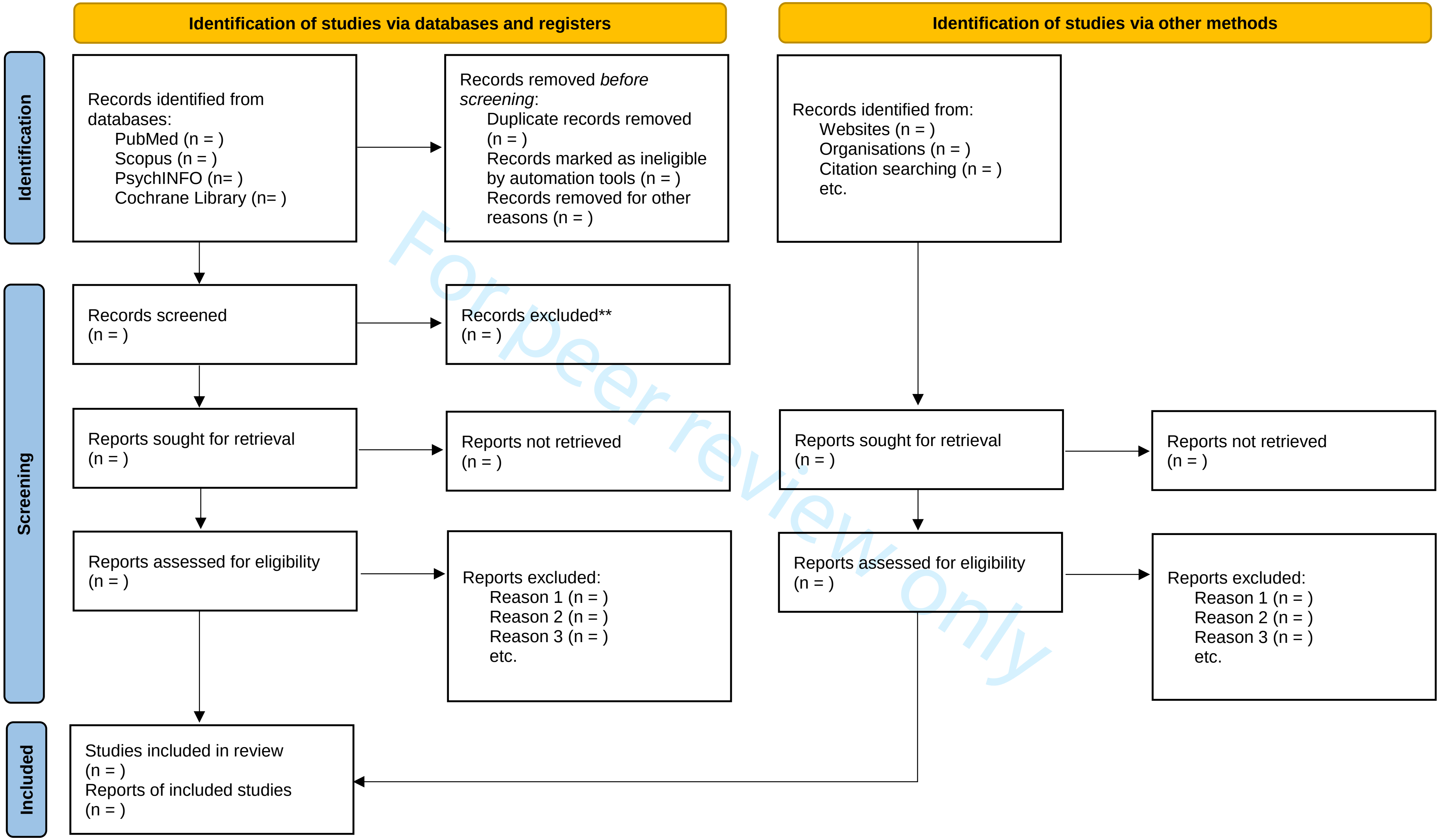

*Consider, if feasible to do so, reporting the number of records identified from each database or register searched (rather than the total number across all databases/registers).
**If automation tools were used, indicate how many records were excluded by a human and how many were excluded by automation tools.

From: Page MJ, McKenzie JE, Bossuyt PM, Boutron I, Hoffmann TC, Mulrow CD, et al. The PRISMA 2020 statement: an updated guideline for reporting systematic reviews. BMJ 2021;372:n71. doi: 10.1136/bmj.n71. For more information, visit: http://www.prisma-statement.org/

For peer review only

Supplementary Table 1. Draft Data Extraction Table.

Element of integrated maternity care	Outcomes					Levels RMIC								Level of evidence
Element of integrated maternity care	Outcome 1. Maternal and neonatal health	Outcome 2. Experiences of professionals	Outcome 3. Experiences of mothers	Outcome 4. Healthcare processes	Healthcare spending	Vertical – Horizontal organization:	Degree of integration	Clinical integration	Professional integration	Organizational integration:	System Integration	Functional enablers:	Normative enablers:	Level of evidence
e.g. mothercouncil														
e.g. multidisciplinary consultation														

For peer review only

Preferred Reporting Items for Systematic reviews and Meta-Analyses extension for Scoping Reviews (PRISMA-ScR) Checklist

SECTION	ITEM	PRISMA-ScR CHECKLIST ITEM	REPORTED ON PAGE #
**TITLE**			
Title	1	Identify the report as a scoping review.	1 (title page)
**ABSTRACT**			
Structured summary	2	Provide a structured summary that includes (as applicable): background, objectives, eligibility criteria, sources of evidence, charting methods, results, and conclusions that relate to the review questions and objectives.	3
**INTRODUCTION**			
Rationale	3	Describe the rationale for the review in the context of what is already known. Explain why the review questions/objectives lend themselves to a scoping review approach.	5-6
Objectives	4	Provide an explicit statement of the questions and objectives being addressed with reference to their key elements (e.g., population or participants, concepts, and context) or other relevant key elements used to conceptualize the review questions and/or objectives.	6
**METHODS**			
Protocol and registration	5	Indicate whether a review protocol exists; state if and where it can be accessed (e.g., a Web address); and if available, provide registration information, including the registration number.	Not applicable (this is the protocol)
Eligibility criteria	6	Specify characteristics of the sources of evidence used as eligibility criteria (e.g., years considered, language, and publication status), and provide a rationale.	6, + table 1 (p10)
Information sources*	7	Describe all information sources in the search (e.g., databases with dates of coverage and contact with authors to identify additional sources), as well as the date the most recent search was executed.	8
Search	8	Present the full electronic search strategy for at least 1 database, including any limits used, such that it could be repeated.	Supplementary File 1; Search Strategy
Selection of sources of evidence†	9	State the process for selecting sources of evidence (i.e., screening and eligibility) included in the scoping review.	9-10
Data charting process‡	10	Describe the methods of charting data from the included sources of evidence (e.g., calibrated forms or forms that have been tested by the team before their use, and whether data charting was done independently or in duplicate) and any processes for obtaining and confirming data from investigators.	12, + Supplementary Table 1; Draft data extraction
Data items	11	List and define all variables for which data were sought and any assumptions and simplifications made.	11

St.Michael's
Inspired Care.
Inspiring Science.

For peer review only - http://bmjopen.bmj.com/site/about/guidelines.xhtml

SECTION	ITEM	PRISMA-ScR CHECKLIST ITEM	REPORTED ON PAGE #
Critical appraisal of individual sources of evidence§	12	If done, provide a rationale for conducting a critical appraisal of included sources of evidence; describe the methods used and how this information was used in any data synthesis (if appropriate).	Not done
Synthesis of results	13	Describe the methods of handling and summarizing the data that were charted.	12
**RESULTS**			
Selection of sources of evidence	14	Give numbers of sources of evidence screened, assessed for eligibility, and included in the review, with reasons for exclusions at each stage, ideally using a flow diagram.	Not applicable – study protocol
Characteristics of sources of evidence	15	For each source of evidence, present characteristics for which data were charted and provide the citations.	Not applicable – study protocol
Critical appraisal within sources of evidence	16	If done, present data on critical appraisal of included sources of evidence (see item 12).	Not applicable – study protocol
Results of individual sources of evidence	17	For each included source of evidence, present the relevant data that were charted that relate to the review questions and objectives.	Not applicable – study protocol
Synthesis of results	18	Summarize and/or present the charting results as they relate to the review questions and objectives.	Not applicable – study protocol
**DISCUSSION**			
Summary of evidence	19	Summarize the main results (including an overview of concepts, themes, and types of evidence available), link to the review questions and objectives, and consider the relevance to key groups.	Not applicable – study protocol
Limitations	20	Discuss the limitations of the scoping review process.	Not applicable – study protocol
Conclusions	21	Provide a general interpretation of the results with respect to the review questions and objectives, as well as potential implications and/or next steps.	Not applicable – study protocol
**FUNDING**			
Funding	22	Describe sources of funding for the included sources of evidence, as well as sources of funding for the scoping review. Describe the role of the funders of the scoping review.	13

JBI = Joanna Briggs Institute; PRISMA-ScR = Preferred Reporting Items for Systematic reviews and Meta-Analyses extension for Scoping Reviews.

* Where *sources of evidence* (see second footnote) are compiled from, such as bibliographic databases, social media platforms, and Web sites.

† A more inclusive/heterogeneous term used to account for the different types of evidence or data sources (e.g., quantitative and/or qualitative research, expert opinion, and policy documents) that may be eligible in a scoping review as opposed to only studies. This is not to be confused with *information sources* (see first footnote).

‡ The frameworks by Arksey and O'Malley (6) and Levac and colleagues (7) and the JBI guidance (4, 5) refer to the process of data extraction in a scoping review as data charting.

§ The process of systematically examining research evidence to assess its validity, results, and relevance before using it to inform a decision. This term is used for items 12 and 19 instead of "risk of bias" (which is more applicable to systematic reviews of interventions) to include and acknowledge the various sources of evidence that may be used in a scoping review (e.g., quantitative and/or qualitative research, expert opinion, and policy document).

For peer review only - http://bmjopen.bmj.com/site/about/guidelines.xhtml

From: Tricco AC, Lillie E, Zarin W, O'Brien KK, Colquhoun H, Levac D, et al. PRISMA Extension for Scoping Reviews (PRISMAScR): Checklist and Explanation. Ann Intern Med. 2018;169:467–473. doi: 10.7326/M18-0850.

For peer review only

St. Michael's
Inspired Care.
Inspiring Science.

For peer review only - http://bmjopen.bmj.com/site/about/guidelines.xhtml

Liebregts search strategies 4 August 2022

Search Strategy for PubMed (4 August 2022)

Search	Query	Results
**#6**	#4 NOT ("Developing Countries"[Mesh] OR "developing countr*"[tiab] OR "developing nation*"[tiab] OR "developing population*"[tiab] OR "developing econom*"[tiab] OR "undeveloped countr*"[tiab] OR "undeveloped nation*"[tiab] OR "undeveloped economy"[tiab] OR "undeveloped economies"[tiab] OR "least developed countr*"[tiab] OR "least developed nation*"[tiab] OR "least developed economy"[tiab] OR "least developed economies"[tiab] OR "less-developed countr*"[tiab] OR "less-developed nation*"[tiab] OR "less-developed population"[tiab] OR "less-developed populations"[tiab] OR "less-developed econom*"[tiab] OR "lesser developed countr*"[tiab] OR "lesser developed nation*"[tiab] OR "lesser developed population"[tiab] OR "lesser developed populations"[tiab] OR "lesser developed economy"[tiab] OR "lesser developed economies"[tiab] OR "under-developed countr*"[tiab] OR "under-developed nation*"[tiab] OR "underdeveloped countr*"[tiab] OR "underdeveloped nation*"[tiab] OR "underdeveloped population*"[tiab] OR "underdeveloped econom*"[tiab] OR "low income countr*"[tiab] OR "middle income countr*"[tiab] OR "low income nation*"[tiab] OR "middle income nation*"[tiab] OR "low income population*"[tiab] OR "middle income population*"[tiab] OR "low income econom*"[tiab] OR "middle income econom*"[tiab] OR "lower income countr*"[tiab] OR "lower income nation*"[tiab] OR "lower income population*"[tiab] OR "lower income economy"[tiab] OR "lower income economies"[tiab] OR "resource limited"[tiab] OR "low resource countr*"[tiab] OR "lower resource countr*"[tiab] OR "low resource nation*"[tiab] OR "low resource population*"[tiab] OR "low resource economy"[tiab] OR "low resource economies"[tiab] OR "underserved countr*"[tiab] OR "underserved nation*"[tiab] OR "underserved population*"[tiab] OR "underserved economy"[tiab] OR "underserved economies"[tiab] OR "under-served country"[tiab] OR "under-served countries"[tiab] OR "under-served nation"[tiab] OR "under-served nations"[tiab] OR "under-served population"[tiab] OR "under-served populations"[tiab] OR "underserved economy"[tiab] OR "underserved economies"[tiab] OR "deprived countr*"[tiab] OR "deprived nation"[tiab] OR "deprived nations"[tiab] OR "deprived population*"[tiab] OR "deprived economy"[tiab] OR "deprived economies"[tiab] OR "poor countr*"[tiab] OR "poor nation*"[tiab] OR "poor population*"[tiab] OR "poor econom*"[tiab] OR "poorer countr*"[tiab] OR "poorer nation*"[tiab] OR "poorer population*"[tiab] OR "poorer econom*"[tiab] OR "lmic"[tiab] OR "lmics"[tiab] OR "lami"[tiab] OR "transitional countr*"[tiab] OR "transitional nation"[tiab] OR "transitional nations"[tiab] OR "transitional econom*"[tiab] OR "transition countr*"[tiab] OR "transition nation*"[tiab] OR "transition econom*"[tiab] OR "low resource setting*"[tiab] OR "lower resource setting*"[tiab] OR "middle resource setting*"[tiab] OR "Third World*"[tiab]) Filters: from 2012 - 2022	**28,250**

For peer review only - http://bmjopen.bmj.com/site/about/guidelines.xhtml

VU VRIJE UNIVERSITEIT AMSTERDAM

**#5**	#4 NOT ("Developing Countries"[Mesh] OR "developing countr*"[tiab] OR "developing nation*"[tiab] OR "developing population*"[tiab] OR "developing econom*"[tiab] OR "undeveloped countr*"[tiab] OR "undeveloped nation*"[tiab] OR "undeveloped economy"[tiab] OR "undeveloped economies"[tiab] OR "least developed countr*"[tiab] OR "least developed nation*"[tiab] OR "least developed economy"[tiab] OR "least developed economies"[tiab] OR "less-developed countr*"[tiab] OR "less-developed nation*"[tiab] OR "less-developed population"[tiab] OR "less-developed populations"[tiab] OR "less-developed econom*"[tiab] OR "lesser developed countr*"[tiab] OR "lesser developed nation*"[tiab] OR "lesser developed population"[tiab] OR "lesser developed populations"[tiab] OR "lesser developed economy"[tiab] OR "lesser developed economies"[tiab] OR "under-developed countr*"[tiab] OR "under-developed nation*"[tiab] OR "underdeveloped countr*"[tiab] OR "underdeveloped nation*"[tiab] OR "underdeveloped population*"[tiab] OR "underdeveloped econom*"[tiab] OR "low income countr*"[tiab] OR "middle income countr*"[tiab] OR "low income nation*"[tiab] OR "middle income nation*"[tiab] OR "low income population*"[tiab] OR "middle income population*"[tiab] OR "low income econom*"[tiab] OR "middle income econom*"[tiab] OR "lower income countr*"[tiab] OR "lower income nation*"[tiab] OR "lower income population*"[tiab] OR "lower income economy"[tiab] OR "lower income economies"[tiab] OR "resource limited"[tiab] OR "low resource countr*"[tiab] OR "lower resource countr*"[tiab] OR "low resource nation*"[tiab] OR "low resource population*"[tiab] OR "low resource economy"[tiab] OR "low resource economies"[tiab] OR "underserved countr*"[tiab] OR "underserved nation*"[tiab] OR "underserved population*"[tiab] OR "underserved economy"[tiab] OR "underserved economies"[tiab] OR "under-served country"[tiab] OR "under-served countries"[tiab] OR "under-served nation"[tiab] OR "under-served nations"[tiab] OR "under-served population"[tiab] OR "under-served populations"[tiab] OR "underserved economy"[tiab] OR "underserved economies"[tiab] OR "deprived countr*"[tiab] OR "deprived nation"[tiab] OR "deprived nations"[tiab] OR "deprived population*"[tiab] OR "deprived economy"[tiab] OR "deprived economies"[tiab] OR "poor countr*"[tiab] OR "poor nation*"[tiab] OR "poor population*"[tiab] OR "poor econom*"[tiab] OR "poorer countr*"[tiab] OR "poorer nation*"[tiab] OR "poorer population*"[tiab] OR "poorer econom*"[tiab] OR "lmic"[tiab] OR "lmics"[tiab] OR "lami"[tiab] OR "transitional countr*"[tiab] OR "transitional nation"[tiab] OR "transitional nations"[tiab] OR "transitional econom*"[tiab] OR "transition countr*"[tiab] OR "transition nation*"[tiab] OR "transition econom*"[tiab] OR "low resource setting*"[tiab] OR "lower resource setting*"[tiab] OR "middle resource setting*"[tiab] OR "Third World*"[tiab])	**58,807**
**#4**	#1 AND #2 AND #3	**63,047**
**#3**	"preconception care"[MeSH Terms] OR "prenatal care"[MeSH Terms] OR "Pregnancy"[MeSH] OR "pregnant women"[MeSH Terms] OR "perinatal care"[MeSH Terms] OR "postnatal care"[MeSH Terms] OR "obstetrics"[MeSH Terms] OR "Midwifery"[Mesh] OR "prenatal care"[tiab] OR Antenatal[tiab] OR Preconception*[tiab] OR Maternal*[tiab] OR Maternity[tiab] OR postnat*[tiab] OR Postpartum[tiab] OR perinat*[tiab] OR Prenatal*[tiab] OR "birth care*"[tiab] OR Pregnan*[tiab] OR Midwive*[tiab] OR midwifery[tiab] OR obstetri*[tiab] OR Neonat*[tiab] OR Newborn*[tiab] OR "New born*"[tiab] OR "birth attendant*"[tiab] OR gynaecolog*[tiab] OR gynecolog*[tiab] OR "natal care*"[tiab]	**1,724,086**

For peer review only - http://bmjopen.bmj.com/site/about/guidelines.xhtml

3/9

**#2**	"maternal health"[MeSH Terms] OR "Pregnancy Outcome"[MeSH] OR "Pregnancy Complications"[Mesh] OR "infant health"[MeSH Terms] OR "Infant Mortality"[Mesh] OR "Morbidity"[Mesh] OR "Mortality"[Mesh] OR "Obstetric Labor Complications"[Mesh] OR "maternal health"[tiab] OR "infant health"[tiab] OR "neonatal health"[tiab] OR "neonate health"[tiab] OR "complications"[sb] OR "complicat*"[tiab] OR "adverse effects"[sb] OR "adverse effect*"[tiab] OR "adverse affect*"[tiab] OR "adversely affect*"[tiab] OR "side effect*"[tiab] OR "side affect*"[tiab] OR "adverse event*"[tiab] OR morbid*[tiab] OR death*[tiab] OR mortalit*[tiab] OR injur*[tiab] OR "adverse outcome*"[tiab] OR "Patient Satisfaction"[MeSH] OR ((Patient*[tiab] OR client*[tiab] OR "Women"[Mesh] OR Woman*[tiab] OR Women*[tiab]) AND (experience*[tiab] OR satisfaction*[tiab])) OR (("health personnel"[MeSH Terms] OR "Nurse Midwives"[Mesh] OR "Obstetrics"[Mesh] OR midwife*[tiab] OR midwiv*[tiab] OR Nurse*[tiab] OR "birth attendant*"[tiab] OR Obstetrician*[tiab] OR gynaecolog*[tiab] OR gynecolog*[tiab] Caregiver*[tiab] OR "care giver*"[tiab] OR Provider*[tiab] OR "health personnel"[tiab] OR "personnel health"[tiab]) AND (experience*[tiab] OR satisfaction*)) OR spending*[tiab] OR expenditure*[tiab] OR cost*[tiab] OR "Referral and Consultation"[Mesh] OR "patient transfer"[MeSH Terms] OR "Medicalization"[Mesh] OR Referral*[tiab] OR Intervention*[tiab] OR Transfer*[tiab] OR medicalization*[tiab] OR medicalisation*[tiab]	**7,660,615**
**#1**	"Intersectoral Collaboration"[Mesh] OR "Models, Organizational"[Mesh] OR "Delivery of Health Care, Integrated"[Mesh] OR "Organization and Administration"[Mesh] OR "integrated care*"[tiab] OR "integrated maternity care*"[tiab] OR "integration of care*"[tiab] OR "Integration of maternity care"[tiab] OR "transmural care"[tiab] OR "continuity of care"[tiab] OR "continuum of care"[tiab] OR "model of care*"[tiab] OR "models of care*"[tiab] OR "care model*"[tiab] OR "care system*"[tiab] OR "risk selection*"[tiab] OR "Shared care"[tiab] OR "Joint care"[tiab] OR "Collaborative care"[tiab] OR Interprofessional*[tiab] OR "Inter professional*"[tiab] OR Collaboration*[tiab] OR Interdisciplinair*[tiab] OR Multidisciplinair[tiab] OR Cooperat*[tiab] OR "co oporat*"[tiab]	**1,952,242**

Search Strategy for Elsevier/Scopus (4 August 2022)

Search	Query	Results
**#7**	#6 AND 2012-2022	**13,145**
**#6**	#5 NOT (conference paper OR conference review)	**22,153**
**#5**	#4 NOT TITLE-ABS-KEY ("developing countr*" OR "developing nation*" OR "developing population*" OR "developing econom*" OR "undeveloped countr*" OR "undeveloped nation*" OR "undeveloped economy" OR "undeveloped economies" OR "least developed countr*" OR "least developed nation*" OR "least developed economy" OR "least developed economies" OR "less-developed countr*" OR "less-developed nation*" OR "less-developed population" OR "less-developed populations" OR "less-developed econom*" OR "lesser developed countr*" OR "lesser developed nation*" OR "lesser developed population" OR "lesser developed populations" OR "lesser developed economy" OR "lesser developed economies" OR "under-developed countr*" OR "under-developed nation*" OR "underdeveloped countr*" OR "underdeveloped nation*" OR "underdeveloped population*" OR "underdeveloped econom*" OR "low income countr*" OR "middle income countr*" OR "low income nation*" OR "middle income nation*" OR "low income population*" OR "middle income population*" OR "low income econom*" OR "middle income econom*" OR "lower income countr*" OR "lower income	**22,727**

Versie: 1

	nation*" OR "lower income population*" OR "lower income economy" OR "lower income economies" OR "resource limited" OR "low resource countr*" OR "lower resource countr*" OR "low resource nation*" OR "low resource population*" OR "low resource economy" OR "low resource economies" OR "underserved countr*" OR "underserved nation*" OR "underserved population*" OR "underserved economy" OR "underserved economies" OR "under-served country" OR "under-served countries" OR "under-served nation" OR "under-served nations" OR "under-served population" OR "under-served populations" OR "underserved economy" OR "underserved economies" OR "deprived countr*" OR "deprived nation" OR "deprived nations" OR "deprived population*" OR "deprived economy" OR "deprived economies" OR "poor countr*" OR "poor nation*" OR "poor population*" OR "poor econom*" OR "poorer countr*" OR "poorer nation*" OR "poorer population*" OR "poorer econom*" OR "lmic" OR "lmics" OR "lami" OR "transitional countr*" OR "transitional nation" OR "transitional nations" OR "transitional econom*" OR "transition countr*" OR "transition nation*" OR "transition econom*" OR "low resource setting*" OR "lower resource setting*" OR "middle resource setting*" OR "Third World*")	
**#4**	#1 AND #2 AND #3	**427,512**
**#3**	TITLE-ABS-KEY ("prenatal care" OR Antenatal OR Preconception* OR Maternal* OR Maternity OR postnat* OR Postpartum OR perinat* OR Prenatal* OR "birth care*" OR Pregnan* OR Midwive* OR midwifery OR obstetri* OR Neonat* OR Newborn* OR "New born*" OR "birth attendant*" OR gynaecolog* OR gynecolog* OR "natal care*")	**25,753**
**#2**	TITLE-ABS-KEY ("maternal health" OR "infant health" OR "neonatal health" OR "neonate health" OR "complicat*" OR "adverse effect*" OR "adverse affect*" OR "adversely affect*" OR "side effect*" OR "side affect*" OR "adverse event*" OR morbid* OR death* OR mortalit* OR injur* OR "adverse outcome*" OR ((Patient* OR client* OR Woman* OR Women*) AND (experience* OR satisfaction*)) OR ((midwife* OR midwiv* OR Nurse* OR "birth attendant*" OR Obstetrician* OR gynaecolog* OR gynecolog* Caregiver* OR "care giver*" OR Provider* OR "health personnel" OR "personnel health") AND (experience* OR satisfaction*)) OR spending* OR expenditure* OR cost* OR Referral* OR Intervention* OR Transfer* OR medicalization* OR medicalisation*)	**2,516,011**
**#1**	TITLE-ABS-KEY ("integrated care*" OR "integrated maternity care*" OR "integration of care*" OR "Integration of maternity care" OR "transmural care" OR "continuity of care" OR "continuum of care" OR "model of care*" OR "models of care*" OR "care model*" OR "care system*" OR "risk selection*" OR "Shared care" OR "Joint care" OR "Collaborative care" OR Interprofessional* OR "Inter professional*" OR Collaboration* OR Interdisciplinair* OR Multidisciplinair OR Cooperat* OR "co oporat*")	**15,178,910**

Search Strategy for Ebsco/APA PsycINFO (4 August 2022)

Search	Query	Results
**#5**	S4 NOT (DE "Developing Countries" OR TI("developing countr*" OR "developing nation*" OR "developing population*" OR "developing econom*" OR "undeveloped countr*" OR "undeveloped nation*" OR "undeveloped economy" OR "undeveloped economies" OR "least developed countr*" OR "least developed nation*" OR "least developed economy" OR "least developed economies" OR "less-developed countr*" OR "less-developed nation*" OR "less-developed population" OR "less-developed populations" OR "less-developed econom*" OR "lesser developed countr*" OR "lesser developed nation*" OR "lesser developed population" OR "lesser developed populations" OR "lesser developed economy" OR "lesser developed economies" OR	**1,019**

For peer review only - http://bmjopen.bmj.com/site/about/guidelines.xhtml

VU VRIJE UNIVERSITEIT AMSTERDAM

5/9

	"under-developed countr*" OR "under-developed nation*" OR "underdeveloped countr*" OR "underdeveloped nation*" OR "underdeveloped population*" OR "underdeveloped econom*" OR "low income countr*" OR "middle income countr*" OR "low income nation*" OR "middle income nation*" OR "low income population*" OR "middle income population*" OR "low income econom*" OR "middle income econom*" OR "lower income countr*" OR "lower income nation*" OR "lower income population*" OR "lower income economy" OR "lower income economies" OR "resource limited" OR "low resource countr*" OR "lower resource countr*" OR "low resource nation*" OR "low resource population*" OR "low resource economy" OR "low resource economies" OR "underserved countr*" OR "underserved nation*" OR "underserved population*" OR "underserved economy" OR "underserved economies" OR "under-served country" OR "under-served countries" OR "under-served nation" OR "under-served nations" OR "under-served population" OR "under-served populations" OR "underserved economy" OR "underserved economies" OR "deprived countr*" OR "deprived nation" OR "deprived nations" OR "deprived population*" OR "deprived economy" OR "deprived economies" OR "poor countr*" OR "poor nation*" OR "poor population*" OR "poor econom*" OR "poorer countr*" OR "poorer nation*" OR "poorer population*" OR "poorer econom*" OR "lmic" OR "lmics" OR "lami" OR "transitional countr*" OR "transitional nation" OR "transitional nations" OR "transitional econom*" OR "transition countr*" OR "transition nation*" OR "transition econom*" OR "low resource setting*" OR "lower resource setting*" OR "middle resource setting*" OR "Third World*") OR AB("developing countr*" OR "developing nation*" OR "developing population*" OR "developing econom*" OR "undeveloped countr*" OR "undeveloped nation*" OR "undeveloped economy" OR "undeveloped economies" OR "least developed countr*" OR "least developed nation*" OR "least developed economy" OR "least developed economies" OR "less-developed countr*" OR "less-developed nation*" OR "less-developed population" OR "less-developed populations" OR "less-developed econom*" OR "lesser developed countr*" OR "lesser developed nation*" OR "lesser developed population" OR "lesser developed populations" OR "lesser developed economy" OR "lesser developed economies" OR "under-developed countr*" OR "under-developed nation*" OR "underdeveloped countr*" OR "underdeveloped nation*" OR "underdeveloped population*" OR "underdeveloped econom*" OR "low income countr*" OR "middle income countr*" OR "low income nation*" OR "middle income nation*" OR "low income population*" OR "middle income population*" OR "low income econom*" OR "middle income econom*" OR "lower income countr*" OR "lower income nation*" OR "lower income population*" OR "lower income economy" OR "lower income economies" OR "resource limited" OR "low resource countr*" OR "lower resource countr*" OR "low resource nation*" OR "low resource population*" OR "low resource economy" OR "low resource economies" OR "underserved countr*" OR "underserved nation*" OR "underserved population*" OR "underserved economy" OR "underserved economies" OR "under-served country" OR "under-served countries" OR "under-served nation" OR "under-served nations" OR "under-served population" OR "under-served populations" OR "underserved economy" OR "underserved economies" OR "deprived countr*" OR "deprived nation" OR "deprived nations" OR "deprived population*" OR "deprived economy" OR "deprived economies" OR "poor countr*" OR "poor nation*" OR "poor population*" OR "poor econom*" OR "poorer countr*" OR "poorer nation*" OR "poorer population*" OR "poorer econom*" OR "lmic" OR "lmics" OR "lami" OR "transitional countr*" OR "transitional nation" OR "transitional nations" OR "transitional econom*" OR "transition countr*" OR "transition nation*" OR	

	"transition econom*" OR "low resource setting*" OR "lower resource setting*" OR "middle resource setting*" OR "Third World*") OR KW("developing countr*" OR "developing nation*" OR "developing population*" OR "developing econom*" OR "undeveloped countr*" OR "undeveloped nation*" OR "undeveloped economy" OR "undeveloped economies" OR "least developed countr*" OR "least developed nation*" OR "least developed economy" OR "least developed economies" OR "less-developed countr*" OR "less-developed nation*" OR "less-developed population" OR "less-developed populations" OR "less-developed econom*" OR "lesser developed countr*" OR "lesser developed nation*" OR "lesser developed population" OR "lesser developed populations" OR "lesser developed economy" OR "lesser developed economies" OR "under-developed countr*" OR "under-developed nation*" OR "underdeveloped countr*" OR "underdeveloped nation*" OR "underdeveloped population*" OR "underdeveloped econom*" OR "low income countr*" OR "middle income countr*" OR "low income nation*" OR "middle income nation*" OR "low income population*" OR "middle income population*" OR "low income econom*" OR "middle income econom*" OR "lower income countr*" OR "lower income nation*" OR "lower income population*" OR "lower income economy" OR "lower income economies" OR "resource limited" OR "low resource countr*" OR "lower resource countr*" OR "low resource nation*" OR "low resource population*" OR "low resource economy" OR "low resource economies" OR "underserved countr*" OR "underserved nation*" OR "underserved population*" OR "underserved economy" OR "underserved economies" OR "under-served country" OR "under-served countries" OR "under-served nation" OR "under-served nations" OR "under-served population" OR "under-served populations" OR "underserved economy" OR "underserved economies" OR "deprived countr*" OR "deprived nation" OR "deprived nations" OR "deprived population*" OR "deprived economy" OR "deprived economies" OR "poor countr*" OR "poor nation*" OR "poor population*" OR "poor econom*" OR "poorer countr*" OR "poorer nation*" OR "poorer population*" OR "poorer econom*" OR "lmic" OR "lmics" OR "lami" OR "transitional countr*" OR "transitional nation" OR "transitional nations" OR "transitional econom*" OR "transition countr*" OR "transition nation*" OR "transition econom*" OR "low resource setting*" OR "lower resource setting*" OR "middle resource setting*" OR "Third World*"))<br>2012 - 2022	
**#4**	#1 AND #2 AND #3	**1,695**
**#3**	DE "Prenatal Care" OR DE "Childbirth Training" OR DE "Pregnancy" OR DE "Adolescent Pregnancy" OR OR DE "Primipara" OR DE "Perinatal Period" OR DE "Postnatal Period" OR DE "Obstetrics" OR DE "Caesarean Birth" OR DE "Midwifery" OR TI("prenatal care" OR Antenatal OR Preconception* OR Maternal* OR Maternity OR postnat* OR Postpartum OR perinat* OR Prenatal* OR "birth care*" OR Pregnan* OR Midwive* OR midwifery OR obstetri* OR Neonat* OR Newborn* OR "New born*" OR "birth attendant*" OR gynaecolog* OR gynecolog* OR "natal care*") OR AB("prenatal care" OR Antenatal OR Preconception* OR Maternal* OR Maternity OR postnat* OR Postpartum OR perinat* OR Prenatal* OR "birth care*" OR Pregnan* OR Midwive* OR midwifery OR obstetri* OR Neonat* OR Newborn* OR "New born*" OR "birth attendant*" OR gynaecolog* OR gynecolog* OR "natal care*") OR KW("prenatal care" OR Antenatal OR Preconception* OR Maternal* OR Maternity OR postnat* OR Postpartum OR perinat* OR Prenatal* OR "birth care*" OR Pregnan* OR Midwive* OR midwifery OR obstetri* OR Neonat* OR Newborn* OR "New born*" OR "birth attendant*" OR gynaecolog* OR gynecolog* OR "natal care*")	**169,395**

For peer review only - http://bmjopen.bmj.com/site/about/guidelines.xhtml

VRIJE UNIVERSITEIT AMSTERDAM

#2	DE "Pregnancy Outcomes" OR DE "Birth" OR DE "Induced Abortion" OR DE "Obstetrical Complications" OR DE "Spontaneous Abortion" OR DE "Morbidity" OR DE "Premorbidity" OR DE "Mortality Rate" OR DE "Mortality Risk" OR DE "Client Satisfaction" OR DE "Client Transfer" OR (("Women"[Mesh] OR "health personnel"[MeSH Terms] OR "Nurse Midwives"[Mesh] OR "Obstetrics"[Mesh]) AND TI(experience* OR satisfaction*) OR AB(experience* OR satisfaction*) OR KW(experience* OR satisfaction*)) OR TI("maternal health" OR "infant health" OR "neonatal health" OR "neonate health" OR "complicat*" OR OR "adverse effect*" OR "adverse affect*" OR "adversely affect*" OR "side effect*" OR "side affect*" OR "adverse event*" OR morbid* OR death* OR mortalit* OR injur* OR "adverse outcome*" OR spending* OR expenditure* OR cost* OR Referral* OR Intervention* OR Transfer* OR medicalization* OR medicalisation*) OR AB("maternal health" OR "infant health" OR "neonatal health" OR "neonate health" OR "complicat*" OR OR "adverse effect*" OR "adverse affect*" OR "adversely affect*" OR "side effect*" OR "side affect*" OR "adverse event*" OR morbid* OR death* OR mortalit* OR injur* OR "adverse outcome*" OR spending* OR expenditure* OR cost* OR Referral* OR Intervention* OR Transfer* OR medicalization* OR medicalisation*) OR KW("maternal health" OR "infant health" OR "neonatal health" OR "neonate health" OR "complicat*" OR OR "adverse effect*" OR "adverse affect*" OR "adversely affect*" OR "side effect*" OR "side affect*" OR "adverse event*" OR morbid* OR death* OR mortalit* OR injur* OR "adverse outcome*" OR spending* OR expenditure* OR cost* OR Referral* OR Intervention* OR Transfer* OR medicalization* OR medicalisation*) OR TI((Patient* OR client* OR OR Woman* OR Women* OR midwife* OR midwiv* OR Nurse* OR "birth attendant*" OR Obstetrician* OR gynaecolog* OR gynecolog* Caregiver* OR "care giver*" OR Provider* OR "health personnel" OR "personnel health") N3 (experience* OR satisfaction*)) OR AB((Patient* OR client* OR OR Woman* OR Women* OR midwife* OR midwiv* OR Nurse* OR "birth attendant*" OR Obstetrician* OR gynaecolog* OR gynecolog* Caregiver* OR "care giver*" OR Provider* OR "health personnel" OR "personnel health") N3 (experience* OR satisfaction*)) OR KW((Patient* OR client* OR OR Woman* OR Women* OR midwife* OR midwiv* OR Nurse* OR "birth attendant*" OR Obstetrician* OR gynaecolog* OR gynecolog* Caregiver* OR "care giver*" OR Provider* OR "health personnel" OR "personnel health") N3 (experience* OR satisfaction*))	**965,418**
#1	DE "Integrated Services" OR TI("integrated care*" OR "integrated maternity care*" OR "integration of care*" OR "Integration of maternity care" OR "transmural care" OR "continuity of care" OR "continuum of care" OR "model of care*" OR "models of care*" OR "care model*" OR "care system*" OR "risk selection*" OR "Shared care" OR "Joint care" OR "Collaborative care" OR Interprofessional* OR "Inter professional*" OR Collaboration* OR Interdisciplinair* OR Multidisciplinair OR Cooperat* OR "co oporat*") OR AB("integrated care*" OR "integrated maternity care*" OR "integration of care*" OR "Integration of maternity care" OR "transmural care" OR "continuity of care" OR "continuum of care" OR "model of care*" OR "models of care*" OR "care model*" OR "care system*" OR "risk selection*" OR "Shared care" OR "Joint care" OR "Collaborative care" OR Interprofessional* OR "Inter professional*" OR Collaboration* OR Interdisciplinair* OR Multidisciplinair OR Cooperat* OR "co oporat*") OR KW("integrated care*" OR "integrated maternity care*" OR "integration of care*" OR "Integration of maternity care" OR "transmural care" OR "continuity of care" OR "continuum of care" OR "model of care*" OR "models of care*" OR "care model*" OR "care system*" OR "risk selection*" OR "Shared care" OR "Joint care" OR "Collaborative care"	**129,901**

VRIJE UNIVERSITEIT AMSTERDAM

	OR Interprofessional* OR "Inter professional*" OR Collaboration* OR Interdisciplinair* OR Multidisciplinair OR Cooperat* OR "co oporat*")	

Search Strategy for Wiley/Cochrane Library (4 August 2022)

Search	Query	Results
**#6**	#5 AND 2012-2022	**2,112**
**#5**	#4 NOT ("developing countr*" OR "developing nation*" OR "developing population*" OR "developing econom*" OR "undeveloped countr*" OR "undeveloped nation*" OR "undeveloped economy" OR "undeveloped economies" OR "least developed countr*" OR "least developed nation*" OR "least developed economy" OR "least developed economies" OR "less-developed countr*" OR "less-developed nation*" OR "less-developed population" OR "less-developed populations" OR "less-developed econom*" OR "lesser developed countr*" OR "lesser developed nation*" OR "lesser developed population" OR "lesser developed populations" OR "lesser developed economy" OR "lesser developed economies" OR "under-developed countr*" OR "under-developed nation*" OR "underdeveloped countr*" OR "underdeveloped nation*" OR "underdeveloped population*" OR "underdeveloped econom*" OR "low income countr*" OR "middle income countr*" OR "low income nation*" OR "middle income nation*" OR "low income population*" OR "middle income population*" OR "low income econom*" OR "middle income econom*" OR "lower income countr*" OR "lower income nation*" OR "lower income population*" OR "lower income economy" OR "lower income economies" OR "resource limited" OR "low resource countr*" OR "lower resource countr*" OR "low resource nation*" OR "low resource population*" OR "low resource economy" OR "low resource economies" OR "underserved countr*" OR "underserved nation*" OR "underserved population*" OR "underserved economy" OR "underserved economies" OR "under-served country" OR "under-served countries" OR "under-served nation" OR "under-served nations" OR "under-served population" OR "under-served populations" OR "underserved economy" OR "underserved economies" OR "deprived countr*" OR "deprived nation" OR "deprived nations" OR "deprived population*" OR "deprived economy" OR "deprived economies" OR "poor countr*" OR "poor nation*" OR "poor population*" OR "poor econom*" OR "poorer countr*" OR "poorer nation*" OR "poorer population*" OR "poorer econom*" OR "lmic" OR "lmics" OR "lami" OR "transitional countr*" OR "transitional nation" OR "transitional nations" OR "transitional econom*" OR "transition countr*" OR "transition nation*" OR "transition econom*" OR "low resource setting*" OR "lower resource setting*" OR "middle resource setting*" OR "Third World*"):ti,ab,kw	**2,121**
**#4**	#1 AND #2 AND #3	**2,156**
**#3**	("prenatal care" OR Antenatal OR Preconception* OR Maternal* OR Maternity OR postnat* OR Postpartum OR perinat* OR Prenatal* OR "birth care*" OR Pregnan* OR Midwive* OR midwifery OR obstetri* OR Neonat* OR Newborn* OR "New born*" OR "birth attendant*" OR gynaecolog* OR gynecolog* OR "natal care*"):ti,ab,kw	**126,263**
**#2**	("maternal health" OR "infant health" OR "neonatal health" OR "neonate health" OR "complicat*" OR "adverse effect*" OR "adverse affect*" OR "adversely affect*" OR "side effect*" OR "side affect*" OR "adverse event*" OR morbid* OR death* OR mortalit* OR injur* OR "adverse outcome*" OR ((Patient* OR client* OR Woman* OR Women*) AND (experience* OR satisfaction*)) OR ((midwife* OR midwiv* OR Nurse* OR "birth attendant*" OR Obstetrician* OR gynaecolog* OR gynecolog* Caregiver* OR "care giver*" OR Provider* OR "health personnel" OR "personnel health") AND (experience* OR satisfaction*)) OR	**837,605**

For peer review only - http://bmjopen.bmj.com/site/about/guidelines.xhtml

VRIJE UNIVERSITEIT AMSTERDAM

	spending* OR expenditure* OR cost* OR Referral* OR Intervention* OR Transfer* OR medicalization* OR medicalisation*):ti,ab,kw	
**#1**	("integrated care*" OR "integrated maternity care*" OR "integration of care*" OR "Integration of maternity care" OR "transmural care" OR "continuity of care" OR "continuum of care" OR "model of care*" OR "models of care*" OR "care model*" OR "care system*" OR "risk selection*" OR "Shared care" OR "Joint care" OR "Collaborative care" OR Interprofessional* OR "Inter professional*" OR Collaboration* OR Interdisciplinair* OR Multidisciplinair OR Cooperat* OR "co oporat*"):ti,ab,kw	**31,496**

For peer review only
